# A Simple Nonviral Method to Generate Human Induced Pluripotent Stem Cells Using SMAR DNA Vectors

**DOI:** 10.3390/genes15050575

**Published:** 2024-04-30

**Authors:** Anna Hartley, Luisa Burger, Cornelia L. Wincek, Lieke Dons, Tracy Li, Annabel Grewenig, Toros Taşgın, Manuela Urban, Alicia Roig-Merino, Mehrnaz Ghazvini, Richard P. Harbottle

**Affiliations:** 1 DNA Vector Laboratory, German Cancer Research Center, 69120 Heidelberg, Germany; a.hartley@dkfz-heidelberg.de (A.H.); a.grewenig@dkfz-heidelberg.de (A.G.); alicia.roig.merino@icloud.com (A.R.-M.); 2 Faculty of Biosciences, Heidelberg University, 69120 Heidelberg, Germany; 3 Erasmus MC iPS Core Facility, Erasmus Medical Centre, 3015 GD Rotterdam, The Netherlandsm.ghazvini@erasmusmc.nl (M.G.)

**Keywords:** S/MAR, SMAR DNA vector, nonviral, iPSC, stem cells, reprogramming

## Abstract

Induced pluripotent stem cells (iPSCs) are a powerful tool for biomedical research, but their production presents challenges and safety concerns. Yamanaka and Takahashi revolutionised the field by demonstrating that somatic cells could be reprogrammed into pluripotent cells by overexpressing four key factors for a sufficient time. iPSCs are typically generated using viruses or virus-based methods, which have drawbacks such as vector persistence, risk of insertional mutagenesis, and oncogenesis. The application of less harmful nonviral vectors is limited as conventional plasmids cannot deliver the levels or duration of the factors necessary from a single transfection. Hence, plasmids that are most often used for reprogramming employ the potentially oncogenic Epstein–Barr nuclear antigen 1 (EBNA-1) system to ensure adequate levels and persistence of expression. In this study, we explored the use of nonviral SMAR DNA vectors to reprogram human fibroblasts into iPSCs. We show for the first time that iPSCs can be generated using nonviral plasmids without the use of EBNA-1 and that these DNA vectors can provide sufficient expression to induce pluripotency. We describe an optimised reprogramming protocol using these vectors that can produce high-quality iPSCs with comparable pluripotency and cellular function to those generated with viruses or EBNA-1 vectors.

## 1. Introduction

Cellular reprogramming has provoked a surge of research and clinical trials, using human induced pluripotent stem cells (iPSCs) for disease and drug modelling as well as regenerative therapies. Takahashi and Yamanaka pioneered the reprogramming of mouse and human cells by retroviral gene transfer [1,2]. However, this method is associated with safety concerns of high oncological risk from insertional mutagenesis. Since then, researchers have developed safer and more efficient reprogramming methods to advance iPSCs toward clinical use. Many of these methods still rely on viruses or viral elements, such as lentiviruses, Sendai virus, and Epstein–Barr virus nuclear antigen (EBNA) vectors [3]. The use of lentiviruses may pose less of a risk than retroviruses, but they can still cause insertional mutagenesis [4]. This risk is particularly relevant in light of the recent directives from the US Food and Drug Administration (FDA), which has called for updated safety warnings on chimeric antigen receptor (CAR) T cell products due to the risk of malignancies linked to retroviral and lentiviral vectors [5,6]. Sendai viral vectors do not integrate into the genome but can linger in reprogrammed cells and restrict their complete differentiation, diminishing their practicality [7,8]. The episomal EBNA plasmid vector system uses the *EBNA-1* gene from the Epstein–Barr virus, which is linked to cancer [9,10,11]. Moreover, viral infection or exposure to viral components can trigger a low-level immune response that persists in reprogrammed cells [12]. Nonviral methods of reprogramming are generally safer but often less practical, involving multiple potentially costly steps. mRNA transfection, for instance, requires multiple daily transfections, making it problematic for clinical manufacturing [13,14]. Protein-based reprogramming is similarly promising but is impeded by the challenge of protein delivery into cells, affecting efficiency [15]. 

Currently, there are two phase III clinical trials using iPSC-derived cells. One, in Japan, aims to treat Parkinson’s disease by transplanting iPSC-derived dopaminergic neurons and has also received approval to start trials in the US [16,17]. The other, in Australia, targets osteoarthritis by injecting iPSC-derived mesenchymal stem cells into the knee joint [18]. Both trials use allogenic iPSCs reprogrammed with the EBNA vector system [19]. Hence, the most clinically viable reprogramming strategy so far involves episomal plasmid vectors which comprise viral components, rather than viral infection.

The aim of this study was to determine whether eliminating all viral components from the reprogramming process is feasible, considering that conventional plasmid-delivered reprogramming factors are incapable of reprogramming cells without repeated transfection [20]. Such a nonviral reprogramming system would carry with it a reduced risk of oncogenesis as well as cellular immune reactions. The EBNA reprogramming vector series (RVS) uses the expression of EBNA-1 and the *oriP* sequence to prolong the expression of reprogramming factors beyond the capability of normal plasmids [21]. The EBNA-1 protein binds to the *oriP* region on each vector, anchoring it to the nuclear matrix and synchronising its replication with the cell cycle [22]. This functionality can be reproduced using a nonviral system, namely, human Scaffold/Matrix Attachment Regions (S/MARs). S/MARs are AT-rich sequences that organise the chromatin in the nucleus. They connect the chromatin to the nuclear matrix and interact with transcription factors and structural proteins to form transcription factories [23,24]. S/MARs coordinate both transcription and genomic replication [25]. Adding an S/MAR sequence to a plasmid vector can keep the vector episomal, leading to stable and inheritable transgene expression without genomic integration (reviewed in this Special Issue by Kreppel and Hagedorn, [26]). We refer to genetic constructs harbouring an S/MAR sequence as SMAR vectors.

There is an increased awareness in the field of gene therapy of the risks associated with the use of viruses and viral vectors and that there may be unwanted cellular consequences to viral transduction [5,27]. Thus, nonviral delivery of therapeutic genes, particularly with human-derived systems, is highly desirable and advantageous for the quality of the resulting cells. SMAR vectors represent a promising alternative to traditional virus-based therapeutic vectors, as they can carry larger therapeutic genes with lower treatment-associated toxicity than many viral vectors. In ground-breaking work, SMAR DNA vectors have been effectively used to genetically engineer haematopoietic progenitor cells with therapeutic genes [28,29,30] and to genetically correct a mouse model of blindness [31]. We built on this work with a recent study that demonstrated that gene augmentation of the 16 kb *USH2A* gene in zebrafish retinas using SMAR vectors, with transgene expression still detectable in vivo after 12 months [32]. Additionally, we have shown that iPSCs can be persistently genetically modified with SMAR vectors, showing stable transgene expression for over 170 days [33]. RNA sequencing analysis of stable SMAR-modified embryonic stem cells shows minimal perturbation to the cells, which were still capable of full differentiation to form transgene-positive chimeric mice upon microinjection into embryos. Transgenes expressed from SMAR vectors also survive the epigenetic remodelling associated with cellular reprogramming by both lentiviral and EBNA reprogramming systems, as well as differentiation into three lineages [33].

To extend this work, in this study, we investigated whether SMAR DNA vectors might also be capable of reprogramming human cells without relying on any viral components. We provide proof of principle that SMAR vectors can reprogram cells, and successfully reprogrammed SMAR iPSCs can be picked and cultured for further use, such as differentiation into functional cell types. We replaced the *EBNA-1-oriP* region in the EBNA vectors with an S/MAR sequence, thus removing all viral sequences from our system, which we expect should lower the immunogenic and oncogenic risks of episomal reprogramming. We describe the modifications to the established EBNA-based reprogramming protocol, which can be used for the nonviral generation of iPSCs using SMAR vectors. Importantly, the protocol developed here is feasible for any research laboratory with typical resources and does not require specialised equipment, such as high-end electroporators. This makes our protocol highly accessible to any group interested in nonviral reprogramming. To facilitate this, we have deposited our vectors in the European Plasmid Repository, where they are available upon request.

## 2. Materials and Methods

### 2.1. Cloning and Bacteria

Chemically competent DH5α bacteria (Life Technologies, Darmstadt, Germany) were grown in Luria–Bertani (LB) media or LB agar plates with the appropriate antibiotic (Ampicillin 100 µg/mL; Kanamycin 30 µg/mL) at 37 °C. Vectors in the SMAR RVS were generated from EBNA RVS vectors and pSMAR [33] using In-Fusion cloning (Takara Bio, Kusatsu, Shiga, Japan) according to the manufacturer’s instructions. pSMAR was digested with AgeI and XhoI, and each reprogramming factor cassette was amplified by PCR from the appropriate EBNA vector using primers (1 to 6) listed in Table S1. The shP53 cassette, including the U6 promoter, was amplified from pCXLE-hO_shP53 using primers 7 and 8 and cloned into both SMAR_hO and pSMAR at the PciI site to generate SMAR_hO_shP53 and SMAR_GFP_shP53. To generate EBNA_GFP-p2A-Puro, pCXLE-hUL was digested with EcoRI to replace the UL cassette with a coGFP-p2A-Puro cassette, amplified from pSMARt [34] using primers 9 and 10. Vectors from the nano RVS were synthesised by Aldevron (Fargo, ND, USA). Plasmid vectors were purified from bacteria using the EndoFree Plasmid Maxi kit (QIAGEN, Hilden, Germany), according to the manufacturer’s instructions, and all plasmid preparations were fully sequenced before transfection into cells. DNA concentrations for transfection were measured on a Qubit 4 (QIAGEN) using the dsDNA Broad Range assay.

### 2.2. Cell Culture

All cells were maintained at 37 °C in a humidified atmosphere with 5% CO_2_. HEK293T cells were maintained in DMEM (4500 mg/L glucose, 2 mM L-glutamine, sodium bicarbonate, without sodium pyruvate) supplemented with 10% FBS (Gibco, Life Technologies Europe, Bleiswijk, The Netherlands) and 1% Pen/Strep (Gibco, Life Technologies Europe, Bleiswijk, The Netherlands). Subculture was performed upon reaching approximately 80% confluence using 0.25% trypsin-EDTA (Sigma-Aldrich, St Louis, MO, USA). Neonatal human dermal fibroblasts (NHDF, newborn male) were obtained from Thermo Fisher Scientific (lot No. 2456041, Carlsbad, CA, USA) and cultured on gelatine-coated dishes (0.1% gelatine in distilled water) under DMEM (4500 mg/L glucose, 2 mM L-glutamine, sodium bicarbonate, without sodium pyruvate) supplemented with 10% FBS (Gibco), 1% Pen/Strep (Gibco) and 1% non-essential amino acids (NEAA, Gibco, Life Technologies Europe, Bleiswijk, The Netherlands). Subculture was performed upon reaching approximately 80% confluence using 0.25% trypsin-EDTA (Sigma-Aldrich). NHDFs were expanded for a maximum of two passages before freezing aliquots for reprogramming. iPSCs were cultured on laminin-coated dishes (recombinant iMatrix Laminin-511 silk E8, Amsbio, Berenkoog, The Netherlands) under StemFit Basic04 Complete Type medium with bFGF (Ajinomoto, Tokyo, Japan) supplemented with 1% Pen/Strep (Gibco). For directed differentiation, culture vessels were coated with Matrigel (Corning, Bedford, MA, USA) instead of laminin. Subculture of iPSCs was performed either as clumps using ReLeSR (Stem Cell Technologies, Vancouver, BC, Canada) or as single cells using StemPro Accutase (Gibco, Life Technologies Europe, Bleiswijk, The Netherlands) according to the manufacturer’s instructions.

### 2.3. Cellular Transfection

HEK293T cells were transfected with jetPEI (Polyplus, Illkirch, France), according to the manufacturer’s instructions, and harvested after 24 h for Western blotting. NHDFs were transfected using the Amaxa II electroporator (Lonza) with the NHDF Nucleofection kit (Lonza, Cologne, Germany) and program P-022 according to the manufacturer’s instructions. Cells were maintained in a medium without antibiotics for 24 h post-transfection.

### 2.4. Reprogramming NHDFs

Reprogramming NHDFs was based on an established protocol described for the EBNA RVS [12]. For each condition, 5 × 10^5^ early-passage NHDFs (p4) were transfected as described with the EBNA RVS (4 vectors), SMAR RVS (3 vectors), or nano RVS (3 vectors). A separate transfection with EBNA-GFP or SMAR-GFP was used to quantify transfection efficiency by microscopy on an Incucyte SX5 (Sartorius). For the EBNA and nano RVS, 2 µg of each vector was transfected, which was defined as 1× vector mass. For the SMAR RVS, up to 10 µg (5×) of each vector was transfected, for a total of 30 µg DNA (see Figure 2). Immediately following transfection, cells were seeded into a gelatinised well of a 6-well plate in supplemented DMEM without antibiotics (DMEM, 10% FCS, 2 mM L-glutamine, 1% NEAA, Gibco) to facilitate recovery. The medium was exchanged 24 h post-transfection to supplemented DMEM with 1% Pen/Strep (Gibco). After two days, cells were transferred to a T75 flask, with subsequent medium changes every other day. On the eighth day, cells were replated at a density of 3.125 × 10^3^ cells/cm^2^ on laminin-coated dishes, and the remaining cells were cryopreserved for future use. Twenty-four hours post-replating, the medium was exchanged to StemFit Basic04 (Ajinomoto) with 1% Pen/Strep (Gibco), and cells were fed on a Monday–Wednesday–Friday schedule until the formation of colonies.

### 2.5. iPSC Colony Picking

Once iPSC colonies reached a sufficient size for picking, they were picked and transferred into fresh wells for expansion. An EVOS XL Core microscope (Life Technologies) was sterilised and placed into a cell culture hood under UV irradiation to ensure sterility. For each colony, 1 well of a 24-well plate was prepared by coating with laminin (recombinant iMatrix Laminin-511 silk E8) and pre-warming StemFit Basic04 medium (Ajinomoto) supplemented with a 10 µm ROCK inhibitor (Y-27632, Biogems, Westlake Village, CA, USA). Subsequently, the iPSC colony was identified under the microscope and scratched using a sterile 200 µL pipette tip to delineate its perimeter from neighbouring fibroblasts. If needed, a cross-hatch pattern was created to facilitate colony detachment. The detached colony fragments were aspirated with a 200 µL pipette and transferred into the prepared well with a pre-warmed medium. The medium was refreshed 24 h post-colony picking to remove the ROCK inhibitor.

### 2.6. Alkaline Phosphatase Staining

Reprogrammed cells were assessed for the stemness marker alkaline phosphatase (AP) expression using the Stemgent Alkaline Phosphatase Staining Kit II (Reprocell, Beltsville, MD, USA), following the manufacturer’s protocol, with the adjustment that only 300 µL of fixing and an AP substrate solution per well of a 12-well plate was used. Images of stained colonies were captured using a Perfection V500 Photo scanner (Epson) and an EVOS XL Core Imaging System (Life Technologies, Carlsbad, CA, USA).

### 2.7. Trilineage Differentiation

The STEMdiff Trilineage Differentiation kit (Stem Cell Technologies, Vancouver, BC, Canada) was used to differentiate iPSCs into cells of three embryonic germ layers, according to the manufacturer’s instructions. Briefly, cells were seeded on Matrigel (Corning) coating and cultured using the medium for endoderm (5 days), mesoderm (5 days), and ectoderm (7 days) differentiation with daily medium changes. Upon completion of differentiation, cells were either fixed for immunofluorescence analysis or subjected to RNA harvesting for qRT-PCR analysis, as outlined below.

### 2.8. NK Cell Differentiation

iPSCs were differentiated into NK cells using the STEMdiff NK Cell Kit (Stem Cell Technologies) according to the manufacturer’s instructions. First, iPSCs were seeded into AggreWell400 plates (Stem Cell Technologies) at a density of 500 cells per microwell to form embryoid bodies (EBs). After 12 days, EBs were dissociated and CD34+ haematopoietic progenitor cells (HPCs) were sorted into a Lymphoid Progenitor Expansion Medium at a density of 7500 cells/96 wells using a BD FACSAria Fusion Flow Cytometer (BD Biosciences, Franklin Lakes, NJ, USA). Following two weeks of lymphoid progenitor expansion, cells were further differentiated into NK cells by culturing them for two weeks in an NK Cell Differentiation Medium. The phenotype of the NK cells was assessed via flow cytometry at the end of the differentiation process using a BD LSRFortessa Cell Analyzer and antibodies listed in Table S4.

### 2.9. PCR for Vector Retention

PCR was conducted on snap-frozen cell pellets to detect reprogramming vector presence. Genomic DNA was extracted from cell pellets using the Phire Direct Tissue PCR kit (Thermo Fisher Scientific, Vilnius, Lithuania), according to the manufacturer’s instructions, for the Dilution and Storage protocol. Samples were diluted 1:50 in water, and 1 µL of the diluted sample was used for PCR using kit-provided internal control primers against *SOX21* and custom primers targeting the bacterial origin of replication (Ori) on plasmid vectors: forward 5′-TTTCCATAGGCTCCGCCCCC-3′; reverse 5′-TTGAGATCCTTTTTTTCTGCGCGTAATCTGC-3′; and product size 589 bp. These primers were designed for a two-step PCR with annealing/extension at 72 °C and ran for 40 cycles. As positive controls, 10 ng purified plasmid DNA was used directly for PCR. PCR products were separated on a 2% agarose gel and imaged using a Fusion SL gel documentation system (Vilber Lourmat).

### 2.10. Microscopy

Brightfield imaging was conducted using an EVOS XL Core Imaging System (Life Technologies). Fluorescent imaging of live HEK293T cells was performed on an Eclipse Ti/X-Cite120Led microscope (Nikon). Immunofluorescence images were acquired using a Stellaris 5 confocal microscope (Leica). For NHDF transfection efficiency estimation, cells were imaged 24–48 h post-transfection using the Adherent Cell-By-Cell module of an Incucyte SX5 (Sartorius) with a 10× objective capturing phase contrast and green fluorescence. Analysis was executed using Incucyte 2020B software with the following segmentation settings: Segmentation Adjustment 2; Hole Fill 1000 µm^2^; Cell Detection Sensitivity 1.5; Cell Contrast 1; Cell Morphology 5; Minimum Area 300 µm^2^; and Fluorescent Background Subtraction Top-Hat No Mask Radius 40 µm. Green cells were defined by a minimum Green Mean Intensity (GCU) established relative to non-transfected cells. Images were processed using Fiji, a distribution of ImageJ [35]. Uneven illumination in brightfield images was corrected by pseudo-flat-field correction. The image was subjected to a Gaussian blur (sigma = 50 pixels) to generate a pseudo-flat-field image, and the original image was divided by this flat field with the Image Calculator plus plugin (k1 = mean flat-field intensity). Manual adjustments were made for white balance and brightness in brightfield images, while display ranges were normalised across all samples for each channel in immunofluorescence images. Scale bars were added using Fiji.

### 2.11. Quantitative Reverse-Transcription Polymerase Chain Reaction (qRT-PCR)

RNA was extracted from iPSCs pre- and post-trilineage differentiation using the ReliaPrep RNA Miniprep kit (Promega, Madison, WI, USA) following the manufacturer’s instructions. cDNA synthesis was carried out using Superscript II RT (Invitrogen, Carlsbad, CA, USA) and oligo (dT) primers (Invitrogen, Carlsbad, CA, USA), according to the manufacturer’s instructions, with 2 µg of RNA per sample. For qRT-PCR, 3 µL of cDNA was combined with 2 µL of PCR buffer, 0.6 µL of 50 mM MgCl_2_, 0.4 µL of dNTPs, 0.4 µL of forward and reverse primers (10µM, see Table S2 for sequences), 0.6 µL of SYBR green (Sigma-Aldrich), 0.08 µL of Platinum Taq DNA polymerase (Thermo Fisher Scientific, Vilnius, Lithuania), and 9.52 µL of sterile water. The reaction was run on a CFX384 qPCR cycler (Bio-Rad, Hercules, CA, USA) with cycling conditions detailed in Table S3. Principal component analysis was conducted using GraphPad Prism 10 software (Dotmatics, Boston, MA, USA).

### 2.12. Western Blotting

Transfected HEK293T cells were collected 24 h post-transfection for Western blotting. After trypsinisation, cells were harvested by scraping in supplemented DMEM, centrifuged at 350 g for 5 min at room temperature, and then lysed using a Cell Lysis Buffer (Cell Signaling Technology, Danvers, MA, USA) supplemented with HALT protease and a phosphatase inhibitor (Thermo Fisher Scientific, Rockford, IL, USA) on ice for a minimum of 20 min. The lysates were centrifuged at 16,000× *g* for 30 min at 4 °C, and the protein-containing supernatant was stored at −80 °C. Protein concentration was determined using the Pierce BCA Protein Assay Kit (Thermo Fisher Scientific, Rockford, IL, USA). Per sample, 20 μg of protein was mixed with 4× Laemmli buffer (BioRad, Hercules, CA, USA) and denatured at 95 °C for 5 min. Samples were electrophoresed on Mini-PROTEAN Gels 4–20% (BioRad, Hercules, CA, USA) in Tris-Glycine buffer (BioRad, Hercules, CA, USA) at 80–120 V for 1–2 h. Protein transfer to the PVDF membrane was performed using the iBlot2 system (Invitrogen) at 20 V for 7 min. The membranes were blocked in 5% milk powder in TBST (0.1% Tween-20) for 1 h, followed by overnight incubation at 4 °C with the primary antibody (see Table S4) diluted in 5% milk in TBST. After washing 3 times for 5 min with TBST, membranes were incubated in a secondary antibody (see Table S5) diluted in 5% milk in TBST for 1 h. Finally, membranes were washed three times again and developed using the Western Lightning Plus ECL reagent (PerkinElmer, Groningen, The Netherlands) in an ECL ChemoCam imager (Intas).

### 2.13. Immunofluorescence

Cells were cultured on 4-well slides with removable frames (Sarsted, Nürnbrecht, Germany) for immunofluorescence, and were first treated with ice-cold methanol for 10 min, followed by a 5 min wash with PBS. They were then permeabilised using 0.1% Triton-X100 in PBS for 10 min at room temperature and washed once with PBS. Subsequently, cells were blocked with 3% BSA/0.1% Tween-20 in PBS (sterile filtered) for 30 min. Cells were incubated in primary antibodies (see Table S4) diluted in a blocking buffer overnight at 4 °C. Slides were washed three times with PBS and incubated with a secondary antibody (see Table S5) in a blocking buffer for 1 h at room temperature. Following this, the excess antibody was removed by three washes with PBS. Finally, cells were mounted using the Vectashield Mounting Medium with DAPI (Vector Laboratories, Newark, NJ, USA) and imaged using a Stellaris 5 confocal microscope (Leica).

### 2.14. Statistical Analysis

Data were analysed with GraphPad Prism 10 (Dotmatics). Statistical tests used are specified in figure legends for each experiment.

## 3. Results

### 3.1. Generation of a Novel Nonviral Reprogramming Platform

**Figure 1:**
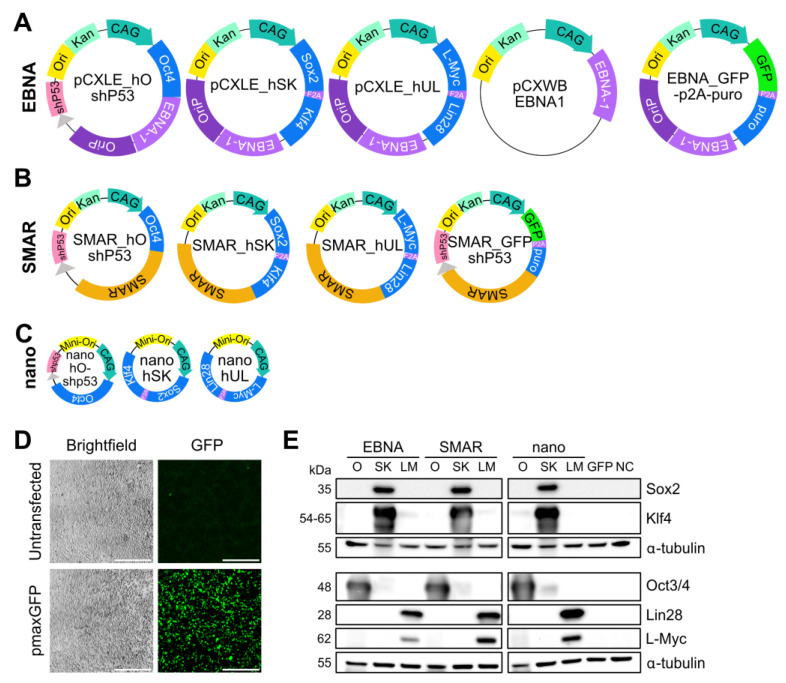
Vector constructs used in this study and their functionality. Schematics of each vector used in this study with representative sizes. (**A**) EBNA series, designed and generated by the Yamanaka group [19]. EBNA-GFP-p2A-puro is a vector for selection not used in reprogramming. (**B**) SMAR series. SMAR_GFP shP53 is a selection vector not used in reprogramming. (**C**) Nano series, with a nanovector backbone and no episomal retention module. (**D**) HEK293T cells were transfected with vectors from the three different reprogramming series and pmaxGFP as a positive control using jetPEI. Cells were imaged 24 hpt using a Nikon fluorescence microscope. The expression of GFP was used as a proxy for successful transfection. Scale = 500 µm. (**E**) Protein lysates from transfected HEK293T cells 24 hpt were assessed for the expression of reprogramming factors Oct3/4 (O), Sox2, Klf4 (SK), Lin28, and L-Myc (LM). GFP and untransfected cells (NCs) were used as negative controls. The expression of proteins was assessed using two identical Western blot membranes; α-tubulin was used as a loading control and is shown for each membrane.

SMAR reprogramming vectors were derived from the established four-vector EBNA plasmid RVS (Figure 1A) by substituting the *EBNA-1-OriP* region with the interferon-β (IFNβ) S/MAR on a traditional plasmid background (Figure 1B). As the S/MAR region requires active transcription for its episomal function, it was cloned in frame with the reprogramming cassette [36]. The fourth vector in the EBNA RVS is an “EBNA boost” plasmid, which is designed to transiently increase the expression of the EBNA-1 protein [11]. S/MAR sequences do not rely on the expression of a protein for episomal retention, making this fourth plasmid unnecessary in the SMAR RVS. Instead, a fourth SMAR vector encoding only coGFP and puromycin resistance was generated to allow for the measurement of transfection efficiency and selection, if required (Figure 1B). Sequences of the SMAR RVS are available from the European Plasmid Repository. We also generated the nano RVS, which encodes each of the reprogramming factors on a minimally sized nanovector background without an S/MAR sequence (Figure 1C, Table S6) [37]. Nanovectors express their transgenes at higher levels than traditional plasmid vectors [33,34]. As the reprogramming process requires a strong but transient expression of ectopic reprogramming factors [38,39,40], we reasoned that a nanovector may give a sufficient expression of reprogramming factors to reprogram cells and improve the safety of these vectors by removing the capacity for vector retention. Vectors were confirmed to be functional by individual transfection into HEK293T cells and Western blotting (Figure 1D,E).

**Figure 2:**
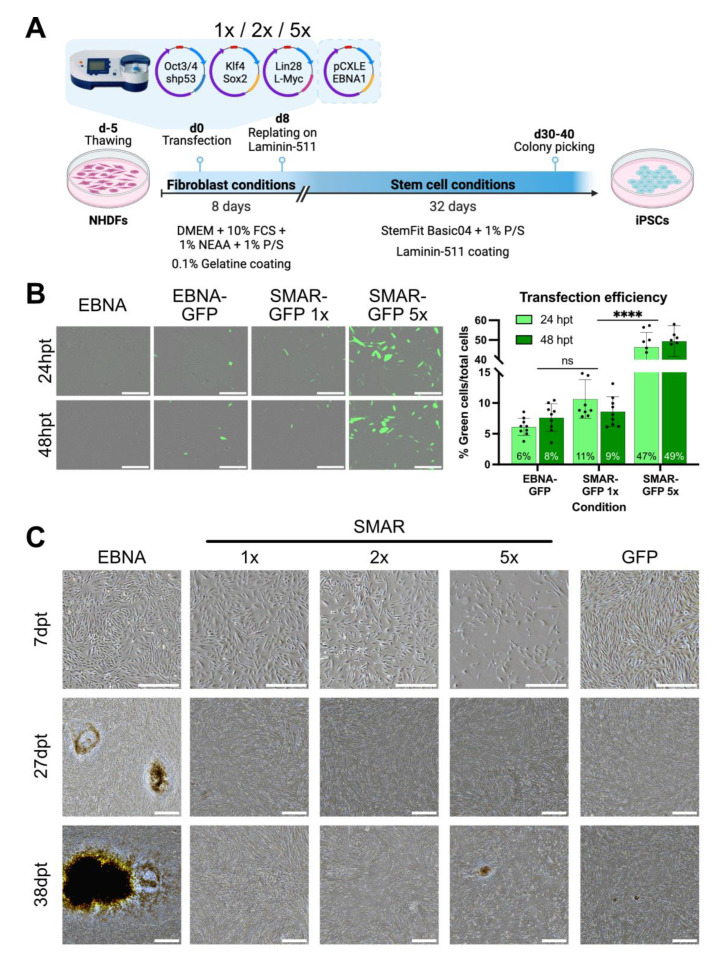
SMAR vectors are capable of reprogramming human dermal fibroblasts. (**A**) Scheme depicting episomal reprogramming by EBNA or SMAR vectors in neonatal human dermal fibroblasts (NHDFs) by transfection with SMAR or EBNA reprogramming vectors at 1×, 2×, or 5× doses of vector by mass. Cells were cultured for 30–40 days post-transfection and monitored for the formation of colonies. (**B**) GFP positivity of transfected NHDFs was quantified 24 h and 48 h post-transfection using an Incucyte SX5. Scale = 500 µm. Data represent mean ± standard deviation; significance was measured by a two-way ANOVA; ns = non-significant; **** = *p* ≤ 0.0001. (**C**) Brightfield images of NHDFs after transfection over time. Scale = 500 µm. Data shows three time points from one representative experiment.

Somatic NHDFs were reprogrammed in a head-to-head comparison of each RVS using an established protocol that robustly yields iPSC colonies from the EBNA RVS (Figure 2A). A separate transfection of cells with EBNA-GFP or SMAR-GFP vectors allowed for the quantification of transfection efficiency (Figure 2B). Following our established protocol, 5 × 10^5^ NHDFs were transfected using the Amaxa II electroporator (Lonza) with 2 µg per vector of each RVS and were then expanded and cultured under fibroblast conditions for 8 days. Cells were then replated and cultured under stem cell conditions until iPSC colonies formed and were monitored by microscopy for up to 40 days post-transfection (Figure 2A and Figure S1A). When cells were transfected with 2 µg of the control GFP vector (1× condition), both vector series showed comparable transfection efficiency (Figure 2B). As expected, the formation of small colonies was visible by 21 days post-transfection in EBNA RVS-transfected cells, which grew to substantial sizes by day 27 post-transfection (Figure 2C). In contrast, cells transfected with the SMAR and nano RVSs showed dramatic morphological changes but no formation of iPSC colonies under the same conditions (Figures S1B and 2C, SMAR1x condition).

To encourage iPSC colony formation using the SMAR RVS, we focussed on improving the transfection efficiency in NHDFs. Increasing the mass of transfected SMAR-GFP DNA by five-fold (10 µg, 5× condition) linearly increased transfection efficiency to approximately 50% (Figure 2B). Thus, we tested iPSC colony formation after transfection with different doses of SMAR RVS (1×, 2×, and 5× the original DNA mass of 2 µg). Despite a slight decrease in the growth of NHDFs transfected with 5× vector dose (10 µg per vector, 30 µg total DNA), stable iPSC colonies formed by day 38 post-transfection, which could be picked and cultured further (Figure 2C, SMAR5x condition). Importantly, this protocol robustly results in SMAR iPSC colonies, which can be picked and cultured further in our hands. As a preliminary indicator of stemness, we assessed the activity of alkaline phosphatase (AP), a membrane-bound hydrolase often expressed at higher levels in pluripotent stem cells than in somatic cells [41]. Colonies formed from EBNA and SMAR5x but not SMAR1x or 2× conditions expressed significant levels of AP (Figure S2).

### 3.2. Characterisation of SMAR iPSCs

**Figure 3:**
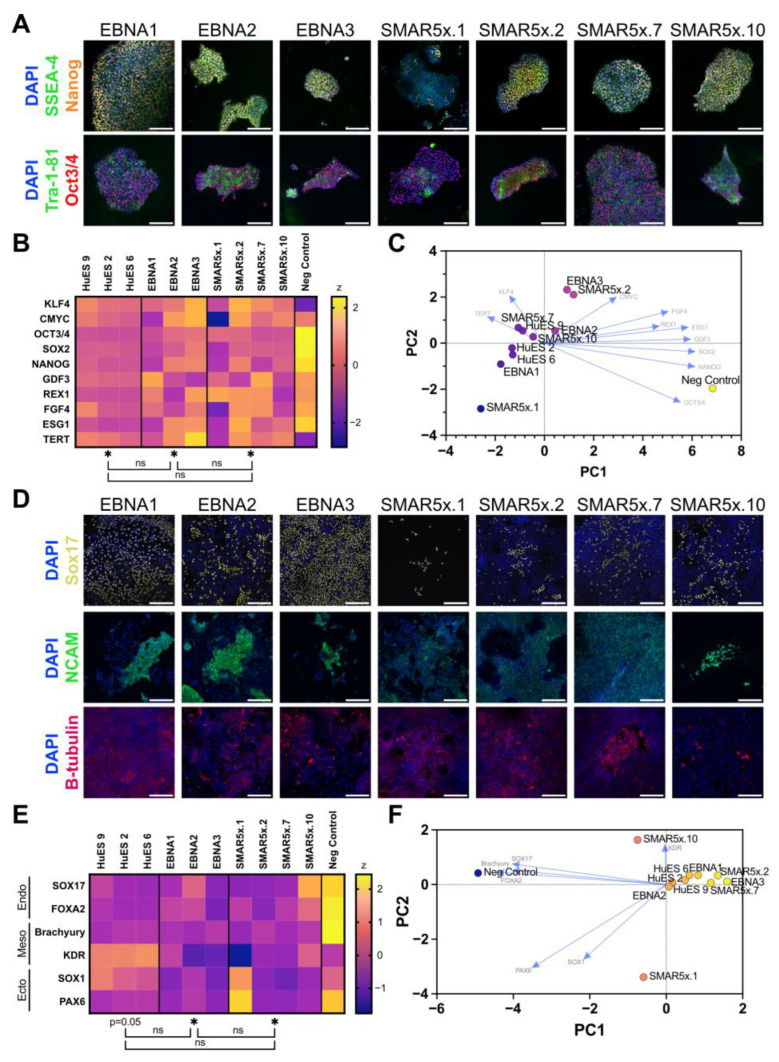
Optimisation of SMAR reprogramming yields high-quality SMAR-iPSCs with similar gene expression to human embryonic stem cells. Three EBNA clones and four SMAR5x clones were analysed (**A**) by immunofluorescence for the expression of stem cell markers SSEA-4 (green), Nanog (orange), Tra-1-81 (green), and Oct3/4 (red), (**B**) by qRT-PCR for their expression of stemness genes compared with three human embryonic stem cell (HuES) lines. Data shown are a z-transformation of the ddCT of gene expression to the negative control fibroblast line. (**C**) Principal component analysis (PCA) of delta Ct expression of stemness genes. Arrows represent the contribution of each gene to the principal component. (**D**) iPSC clones were differentiated into cells of three distinct lineages and analysed by immunofluorescence for the expression of lineage-specific factors. Sox17 (yellow) was used as an endoderm marker, NCAM (green) as a mesoderm marker, and β-tubulin III (magenta) as an ectoderm marker. (**E**) qRT-PCR for lineage-specific markers after directed differentiation of iPSC lines compared with HuES lines. Data shown are a z-transformation of the ddCT of gene expression to the negative control fibroblast line. (**F**) Principal component analysis (PCA) of delta Ct expression of lineage-specific genes. Arrows represent the contribution of each gene to the principal component. Scale = 200 µm. Heatmap data represent mean values, and significance was measured for each group compared to negative control fibroblasts by a one-sample *t*-test assuming 0 as the theoretical mean or between groups (indicated by brackets) by a one-way ANOVA; ns = non-significant; * = *p* ≤ 0.05.

iPSC colonies generated using the protocol developed above were picked for further culture. From a single experiment, four EBNA clones were picked, all of which survived, and ten SMAR5x clones were picked, eight of which survived and one of which reverted to a fibroblast-like morphology. For further characterisation, four SMAR5x iPSC clones were compared with three EBNA clones. We examined the expression of the stemness markers SSEA-4, Nanog, Tra-1-81, and Oct3/4 by immunofluorescence (Figure 3A), only one of which (Oct3/4) is expressed by the reprogramming vectors. All clones tested showed the expression of each stemness marker. Expression analysis by qRT-PCR of a wider range of marker genes in these EBNA and SMAR clones indicated transcriptional profiles similar to three independent human embryonic stem cell (HuES) lines and were strongly divergent to negative control fibroblast cells (Figure 3B,C).

A fundamental property of iPSCs is their pluripotency, which can be evaluated by their capacity to differentiate into cells of the three germ layers. Cellular pluripotency can be assessed either by teratoma formation or in vitro differentiation assays [42,43]. To minimise animal usage, we chose to subject SMAR and EBNA iPSCs to directed tri-lineage differentiation in vitro and stained these cultures for lineage-specific markers Sox17 (endoderm), NCAM (mesoderm), and β-tubulin III (ectoderm) (Figure 3D). All clones tested successfully completed differentiation into each germ layer, as evidenced by positive staining for each marker. The differentiation capacity of these iPSCs varied between clones, including some SMAR clones showing improved differentiation into the mesoderm lineage compared with the EBNA clones tested (Figure 3D, SMAR5x.2, SMAR5x.7). Expression analysis by qRT-PCR again showed similarity between differentiated SMAR iPSCs and differentiated HuES cell lines (Figure 3E), with a tight clustering of the majority of EBNA and SMAR5x iPSC lines with HuES lines in principal component analysis and a clear separation of control fibroblasts (Figure 3F). Thus, the optimised SMAR reprogramming protocol developed in this study can generate high-quality, pluripotent SMAR iPSCs.

**Figure 4:**
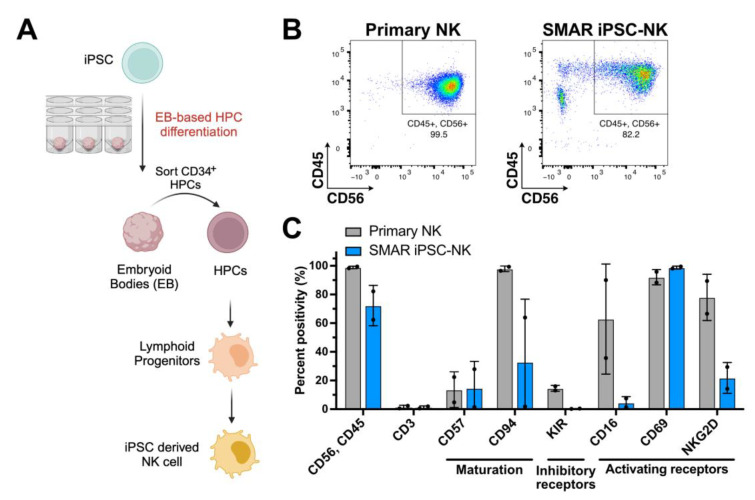
SMAR iPSCs can be differentiated into phenotypic natural killer (NK) cells. (**A**) Scheme depicting the differentiation process from iPSC to NK cells. CD34+ haematopoetic progenitor cells (HPCs) were generated from SMAR iPSCs by embryoid body (EB) formation and cultured for 12 days, after which CD34+ cells were sorted and further differentiated into lymphoid progenitor cells and NK cells in a two-step process over 28 days. (**B**) The expression of major NK cell marker CD56 and leukocyte marker CD45 in primary NK cells and SMAR iPSC-derived NK cells as measured by flow cytometry. (**C**) The expression of a panel of NK cell markers in primary NK cells and SMAR iPSC-derived NK cells as measured by flow cytometry, including maturation markers (CD57, CD94), inhibitory receptors (Killer Inhibitory Receptors, KIRs), and activating receptors (CD16, CD69, NKG2D). Data shown are from two independent experiments.

To demonstrate a functional use case for SMAR iPSCs, we targeted their differentiation into natural killer (NK) cells (Figure 4). iPSCs provide a versatile source for NK cell production in clinical settings due to their unlimited capacity to divide and their comparative amenability to genetic engineering, as opposed to mature NK cells. iPSC-derived NK cells expressing tumour antigen-targeting CARs are currently used in five ongoing clinical trials against different cancer entities [44]. We differentiated SMAR iPSCs in two independent experiments and compared the phenotype of our iPSC-derived NK cells to primary NK cells derived from human peripheral blood by flow cytometry. NK cells are characterised by the expression of markers CD45 and CD56, as well as an absence of the T cell marker CD3. In both experiments, high percentages of CD45+/CD56+/CD3- NK cells were obtained, demonstrating the ability of SMAR iPSCs to differentiate into mature cell types (Figure 4B,C). Furthermore, we analysed the expression of inhibitory and activating receptors on SMAR iPSC-derived NK cells (Figure 4C). We could detect a high expression of the activating receptor CD69 and some expression of CD16 and NKG2D, while inhibitory KIRs were mostly absent, indicating an active NK phenotype.

### 3.3. Generation of Vector-Free iPSCs

**Figure 5:**
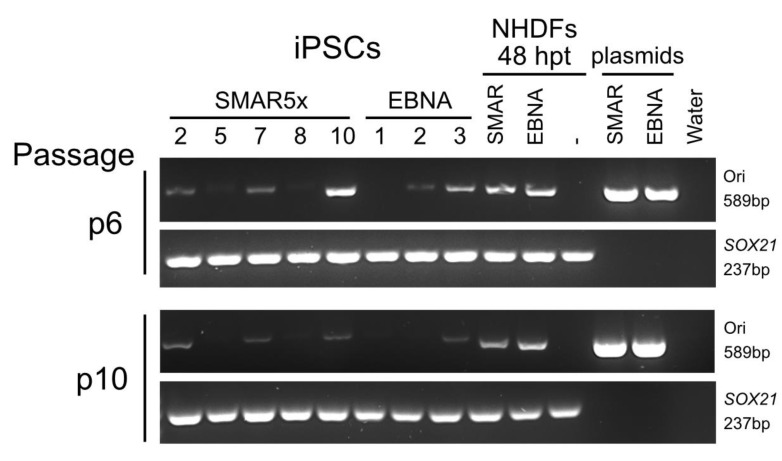
SMAR iPSCs are able to lose SMAR vectors after reprogramming. The presence of the reprogramming vectors was assessed by PCR on genomic DNA for the plasmid origin of replication (Ori, 589 bp) at passage 6 and passage 10 after iPSC colony establishment. A 237 bp fragment corresponding to the *SOX21* gene was used as an internal control to confirm the presence of a genomic DNA template. Five SMAR and three EBNA clones were assessed for the presence of the vectors. Data are representative of three independent replicates.

One major aim of SMAR-based reprogramming is to improve the safety of the reprogramming process with respect to genomic integrity. Ideally, iPSCs for clinical application should be vector free and factor free, leading to the aim to remove reprogramming vectors after the completion of the process. It has previously been reported that approximately 70% of EBNA-reprogrammed cells passively lose their episomal vectors by passage 20, with a loss of approximately 2–8% of vector per cell division [19,45]. Importantly, this loss of vector does not cause cell-fate reversion, as the expression of endogenous pluripotency genes remains stable in vector-free iPSCs [1,46]. We, therefore, assessed the iPSC clones produced in this study for the continued presence of the reprogramming vectors to compare EBNA and SMAR vector retention. The presence of at least one reprogramming vector in iPSC clones was detected by PCR on the bacterial origin of replication present on all EBNA and SMAR vectors (Figure 1A,B, yellow “Ori” segments). We chose to test our iPSC clones at passages six and ten after picking. The presence of episomal vectors was already undetectable in two SMAR clones and one EBNA clone at passage six, while a second EBNA clone lost detectable vectors by passage ten (Figure 5). This indicates that while SMAR vectors have the potential for passive loss after reprogramming by passage ten (40% of clones tested), this loss may be less efficient than the EBNA vectors (66% of clones tested).

## 4. Discussion

In this study, we introduce a novel method for fully reprogramming healthy human fibroblasts using SMAR DNA vectors. The process of generating iPSCs with the SMAR RVS takes marginally longer than with the EBNA RVS, yet the reprogramming efficiency remains within a practical range for routine use. iPSCs reprogrammed with the SMAR RVS are phenotypically comparable to those reprogrammed with the EBNA RVS and exhibit protein and gene expression patterns similar to HuES cells. We also show that SMAR-iPSCs can be differentiated into clinically relevant functional cell types, such as NK cells. The protocol outlined here employs a commonly available laboratory electroporator, making it a readily adoptable technique for any research facility. While successful reprogramming using SMAR vectors is similarly stochastic as for EBNA vectors, the process is robust and, in our hands, generates bona fide iPSCs from each experiment conducted. For ease of handling, this protocol is designed to accommodate a typical laboratory schedule, with culture medium changes solely on Mondays, Wednesdays, and Fridays with no weekend hands-on time. We placed a particular focus on the accessibility of our protocol, as technically difficult protocols, such as mRNA transfection, are more likely to be rejected due to a failure to establish the methods in laboratories. In contrast, our protocol is akin to the well-established EBNA-based reprogramming, which has proven to have high success rates in research laboratories and facilities globally [47].

In contrast to the SMAR RVS, we found that the nano RVS, which lacks an S/MAR motif, was unable to reprogram fibroblasts, reaffirming the importance of the sustained expression of the reprogramming factors in this process (Figure S1) [20]. Despite enhancements in vector expression and retention over conventional plasmids [33,34], the nano RVS’s performance suggests that a nanovector backbone cannot fully compensate for the absence of an episomal element. Indeed, the reprogramming process has been segmented into distinct phases known as initiation, maturation, and stabilisation [48], and the sustained expression of reprogramming factors through this maturation phase is clearly required for successful reprogramming [49,50]. The key threshold appears to be around days 15–17, before which the removal of OKSM expression results in the reversion of cell fate [49]. Thus, it is clear from this work that an episomal retention factor, such as EBNA-1/*OriP* or an S/MAR motif, is necessary and sufficient to drive OKSML expression through this critical threshold to allow reprogramming from a single plasmid transfection.

Previous work with SMAR vectors has demonstrated their ability to label cells for in vivo tracing or to introduce therapeutic genes with non-integrative but stable gene expression [32,51,52]. As an example, we have recently shown that SMAR vectors allow for efficient transfection and modification of primary human T cells with CAR constructs [34]. Importantly, proteogenomic analysis of SMAR CAR-T cells shows lower immune stimulation and a more naïve phenotype than lentivirus-derived T cells, leading to an improved CAR-T efficacy in vitro and in vivo. We have also demonstrated a stable expression of SMAR-delivered transgenes in iPSCs and mouse embryonic stem cells for periods of up to 170 days [33]. Importantly, in this previous study, we showed by RNA sequencing that the addition of SMAR vectors to stem cells causes minimal perturbations to the cellular transcriptome [33]. In contrast to these studies, the results presented here represent a completely new application of SMAR vectors as a tool to facilitate epigenetic remodelling, instead of the delivery of a therapeutic transgene. This new application of SMAR vectors carries with it a new set of challenges and goals.

The ultimate aim for clinical-grade iPSC manufacturing is to make cells that are genomically scarless and do not retain any reprogramming vectors or factors. In this study, only two of five (40%) SMAR iPSC clones tested lost detectable vector levels by passage ten. This result suggests that the SMAR RVS has the capacity for spontaneous loss in iPSCs through passaging, as also observed with the EBNA RVS [19,47]. Indeed, it would be informative to evaluate the kinetics of vector loss in SMAR iPSCs, as has been described for EBNA vectors [45]. However, the retention of SMAR vectors in three of five clones is in agreement with our previous findings that established SMAR vectors can persist for extended periods in stem cells without selection [33]. Indeed, it is conceivable that the reprogramming process itself selects for iPSC colonies capable of maintaining vectors over those that are incompletely or unsuccessfully reprogrammed. Thus, the vector design requirements for this new application of SMAR vectors differ from those for stable therapeutic transgene expression. The strategic use of promoters could be employed to encourage vector loss upon the establishment of stable iPSCs. SMAR vectors are typically lost when silenced [53], so cell state-dependent promoters that are susceptible to epigenetic silencing could be used. These can support high expression levels of reprogramming factors early on but become silenced as endogenous OKSM expression is triggered, which would lead to a passive loss of the vector [40]. This is a strategy that is also employed in lentiviral reprogramming and could result in a higher yield of vector-free SMAR iPSCs [46].

As S/MAR sequences can drive episomal plasmid retention without any protein expression, the fourth “boost” plasmid in the EBNA RVS becomes unnecessary in the SMAR RVS. Thus, the SMAR RVS only requires the co-transfection of three vectors for efficient reprogramming, compared to four in the EBNA RVS. Additionally, the SMAR vectors developed here are smaller than the EBNA RVS (Figure 1A,B). These features give the SMAR RVS the potential for higher co-transfection efficiencies than the EBNA RVS. In this study, we found that a transfection efficiency of 50% was sufficient to reprogram NHDFs using the SMAR RVS, although this dose of reprogramming factors also hindered cell growth somewhat after transfection (Figure 2C). Thus, this system can still be further fine tuned to find the optimal dose of vectors to reprogram cells with minimal stress. In addition, the use of other methods of transfection, such as more modern electroporation instruments, may improve transfection efficiencies, thus improving the reprogramming efficiency of the SMAR RVS. Given a sufficiently efficient transfection protocol, it could be advantageous to combine all transgenes on a single cassette, mirroring the approach typically adopted in lentiviral reprogramming [46]. This would ensure that each successfully transfected cell acquires all requisite factors for reprogramming.

While we provide a strong proof-of-principle here that SMAR vectors can be used for nonviral plasmid-based reprogramming, there is still scope for improvement of the SMAR reprogramming system beyond vector design. Recent discoveries have identified a range of small molecules that enhance reprogramming efficiency by modulating critical cellular pathways, such as inhibitors of histone deacetylation or DNA methylation like valproic acid, or 5-azacytidine [54]. Vitamin C has also been demonstrated to increase reprogramming efficiency in both mouse and human cells by mitigating senescence [55]. Indeed, the combined inhibition of specific signalling pathways, including MEK and TGFβ, has already been successfully employed to enhance the reprogramming efficiency of the EBNA RVS [56]. The use of small molecules to improve reprogramming by the SMAR RVS has not yet been explored but may, in the future, lead to higher efficiencies of reprogramming and practicality for use.

Improving the safety of the reprogramming process and the subsequent cell product is a continuous effort in the field, including by Shin Yamanaka’s team, who have recently refined the Sendai virus reprogramming system for more thorough and efficient viral elimination post-reprogramming [8]. Other advances include the use of synthetic linear doggybone DNA to reprogram human cells; work that shed light on the fact that the extraneous expression of EBNA-1 can induce ROS-mediated DNA damage, and cells reprogrammed with the EBNA vector system exhibit a heightened immune response compared to those reprogrammed with nonviral DNA [12]. These findings underscore the importance of virus-free reprogramming methods. The SMAR system presented here, however, stands out for its scalability, offering a cost-effective and straightforward production process for SMAR vectors without the need for post-purification processing as shown previously [34]. Early work by Okita and colleagues established that cells could be reprogrammed through four consecutive transfections using conventional plasmids [57]. Our SMAR system further streamlines this process by adding the nonviral episomal retention of vectors, rendering a single transfection sufficient for reprogramming, which is particularly beneficial for compliance with current good manufacturing practice (cGMP) standards. Further improvements to the SMAR reprogramming system, including ensuring the loss of vector after iPSC colony establishment, and the validation of differentiated cells in in vivo models should establish the SMAR RVS as a viable option for clinical-grade nonviral reprogramming.

## Data Availability

The data presented in this study are available upon request from the corresponding author.

## Supplementary Materials

The following supporting information can be downloaded at https://www.mdpi.com/article/10.3390/genes15050575/s1, Figure S1: Nanovectors are not capable of fully reprogramming NHDFs without episomal maintenance; Figure S2: Colonies formed from EBNA and SMAR5x transfection express alkaline phosphatase; Table S1: List of cloning primers used in this study; Table S2: List of qRT-PCR primers used in this study; Table S3: Cycling conditions for qRT-PCRs to assay iPSC pluripotency and differentiation; Table S4: List of primary antibodies and their dilutions used in this study; Table S5: List of secondary antibodies and their dilutions used in this study; Table S6: List of reprogramming vector series used in this study and their properties.
