# Supplementary material for: A Simple Nonviral Method to Generate Human Induced Pluripotent Stem Cells Using SMAR DNA Vectors
Source: Genes (Basel). 2024 Apr 30;15(5):575. doi: 10.3390/genes15050575 (PMC11121542; doi:10.3390/genes15050575)

Original Western blotting images used in Figure 1E. Two identical membranes were prepared and sequentially blotted for each marker. Images from each membrane are stacked vertically in the order in which they were stained and boxes indicate areas shown in the main figure.

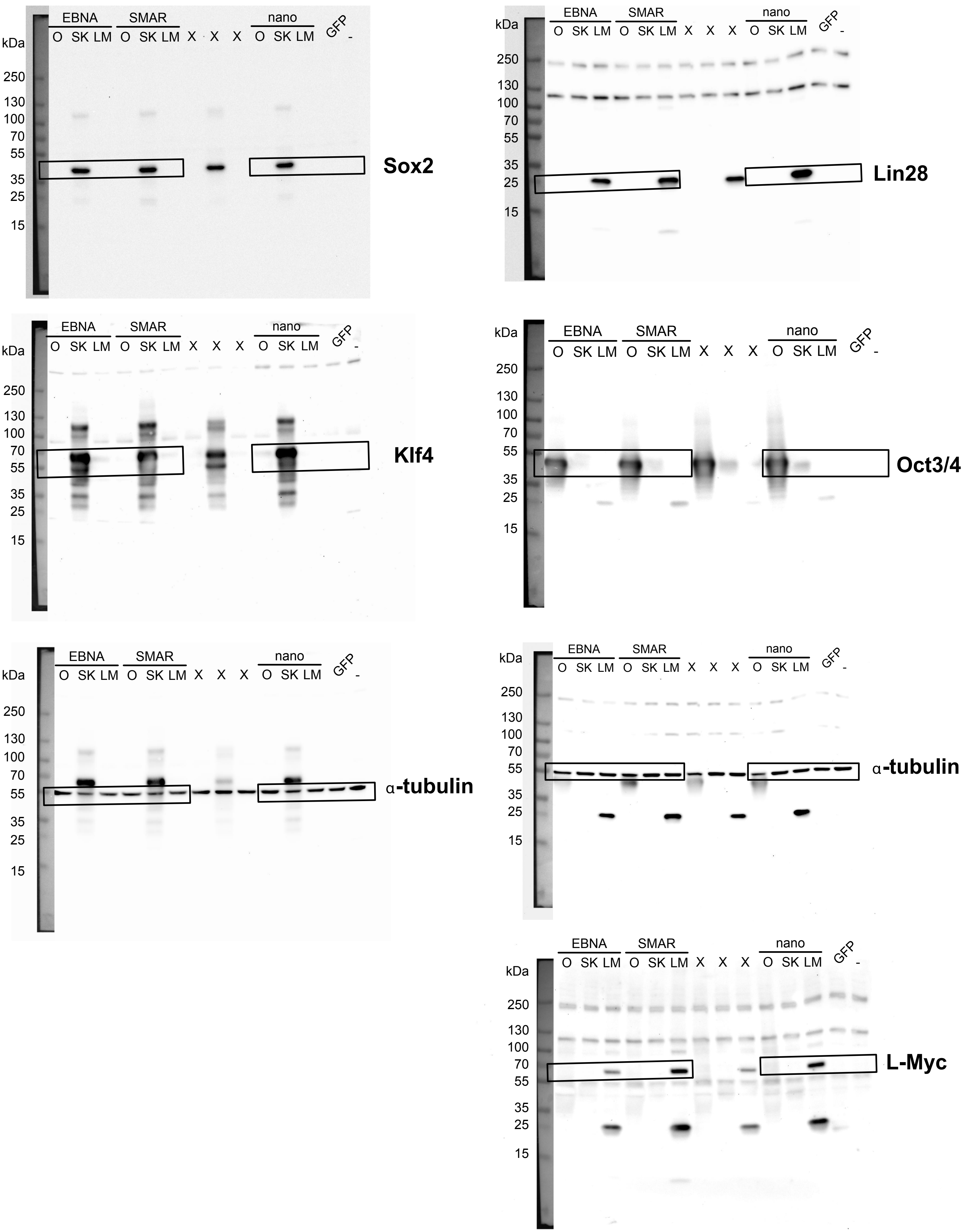

Original DNA gel electrophoresis images used in Figure 5

iPSCs passage 6

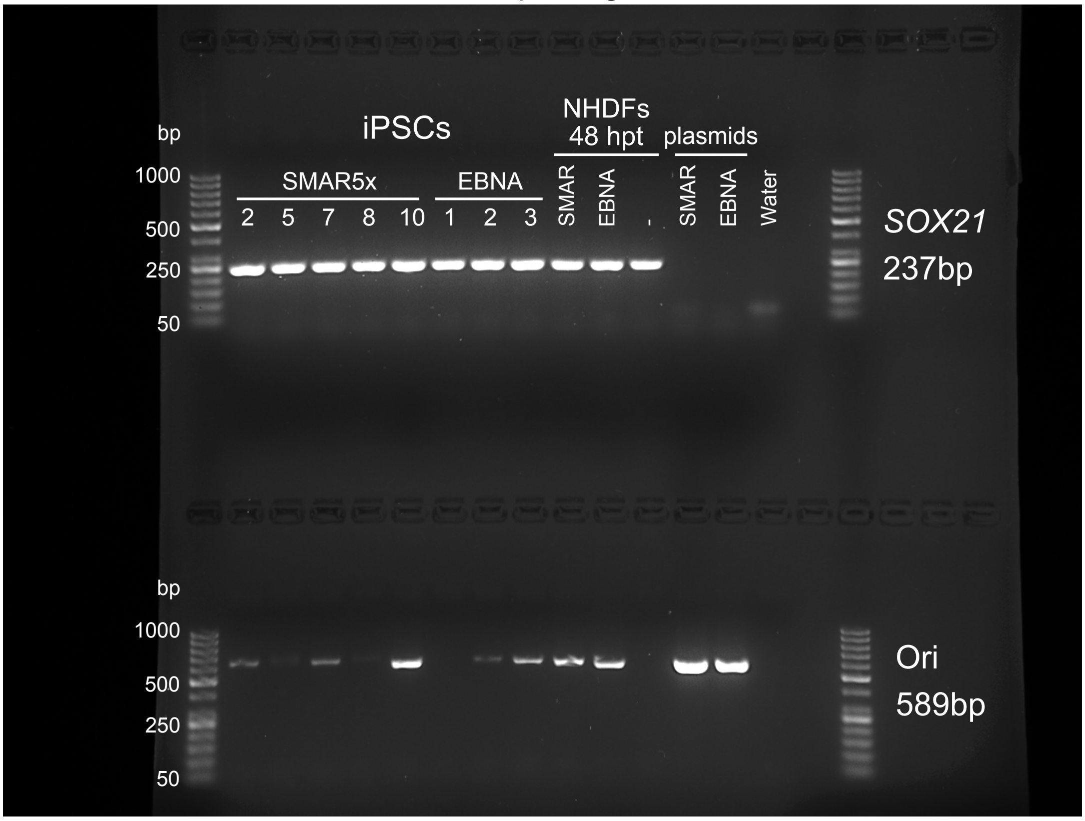

iPSCs passage 10

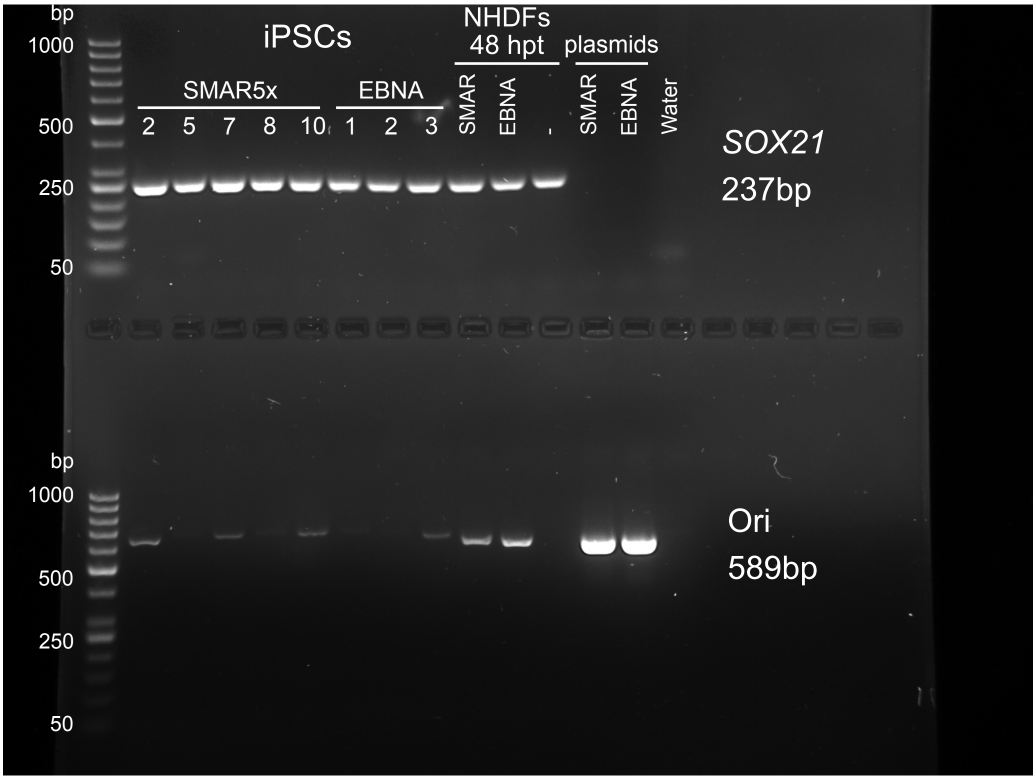

Further two replicates of DNA gel electrophoresis after PCR for vector retention at passage 10 (Figure 5)

Each replicate involved independent passaging of iPSC clones after passage 4 and PCR on cell extracts at passage 10

iPSCs passage 10 rep 2

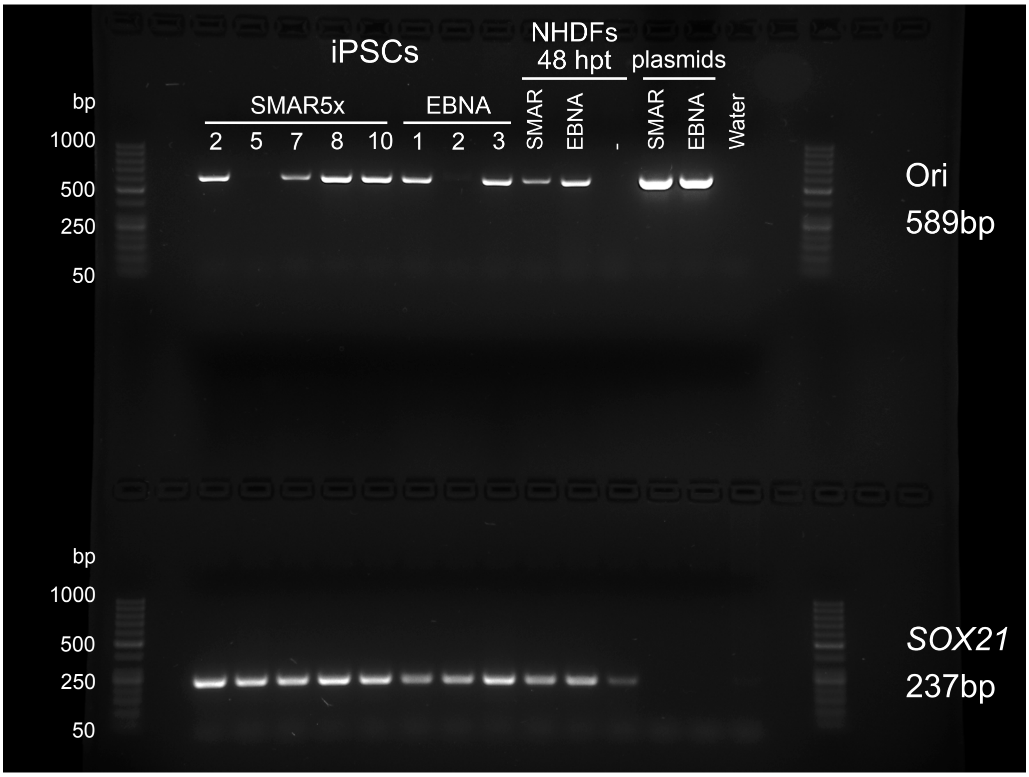

iPSCs passage 10 rep 3

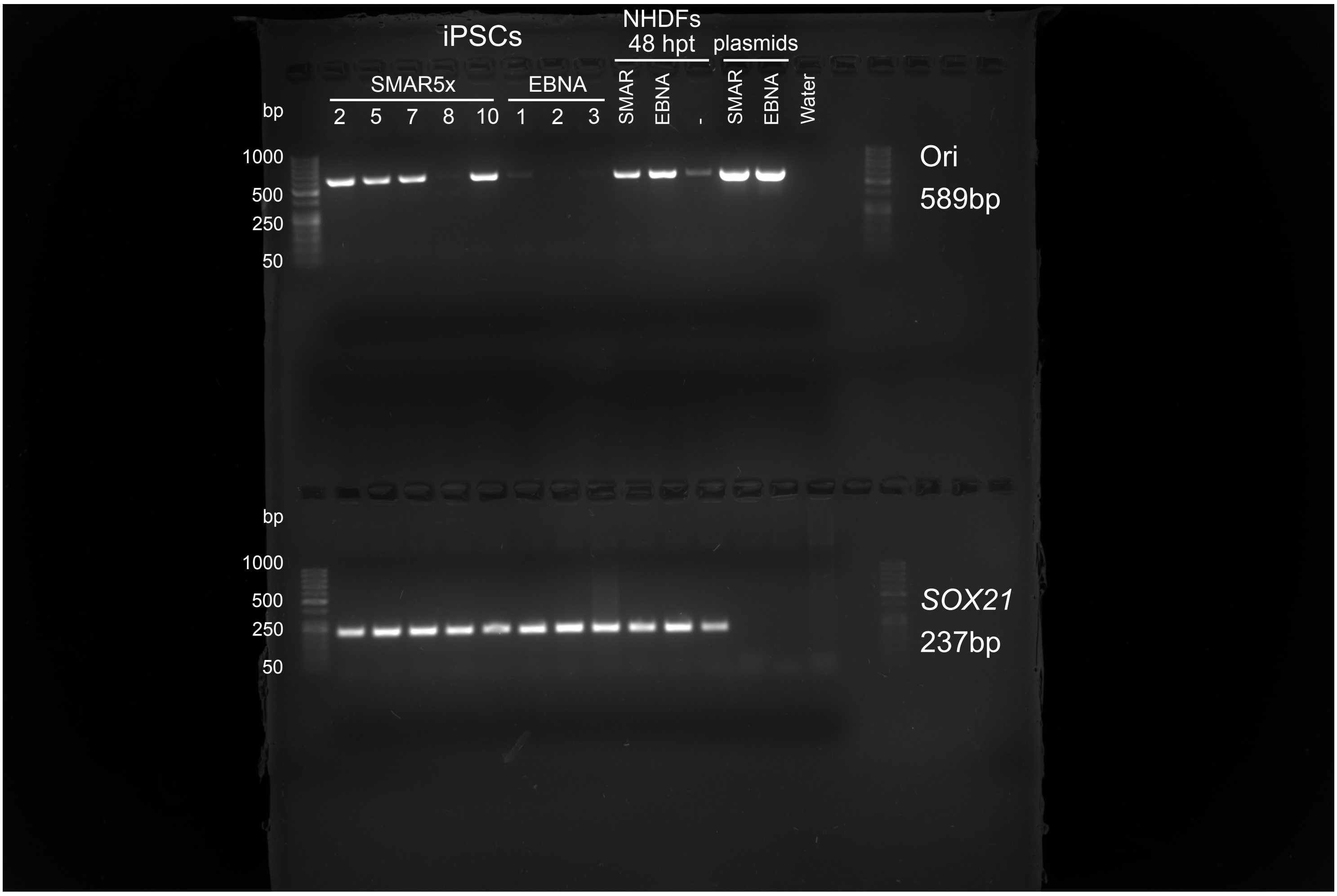

Uncropped, uncorrected fluorescence and brightfield microscopy images used in Figure 1D

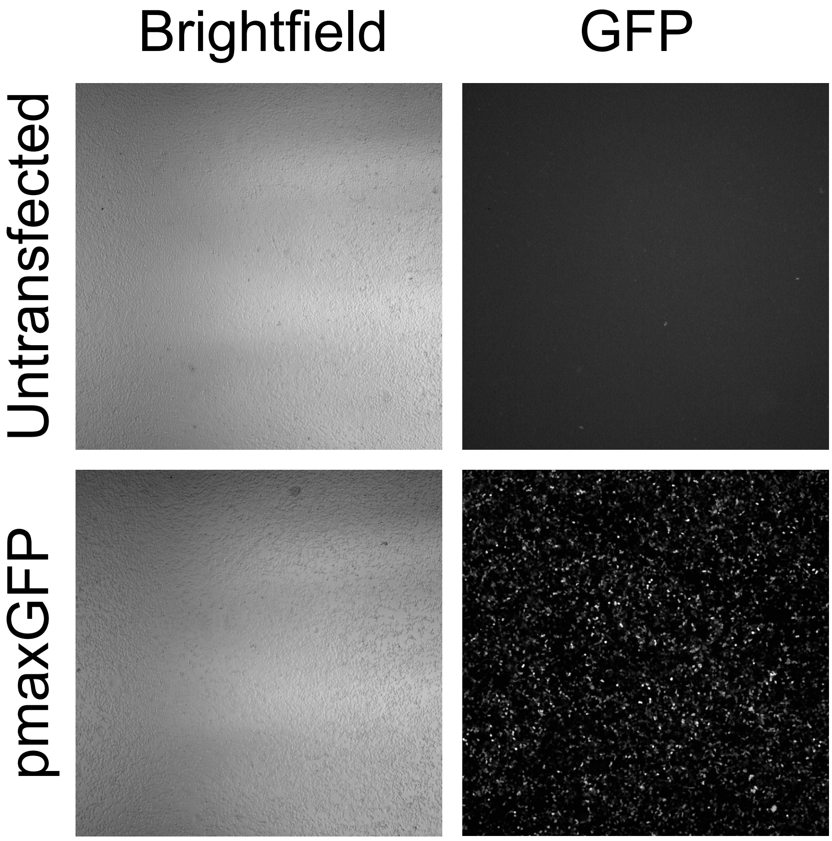

Uncropped, uncorrected fluorescence and brightfield microscopy images used in Figure 2B

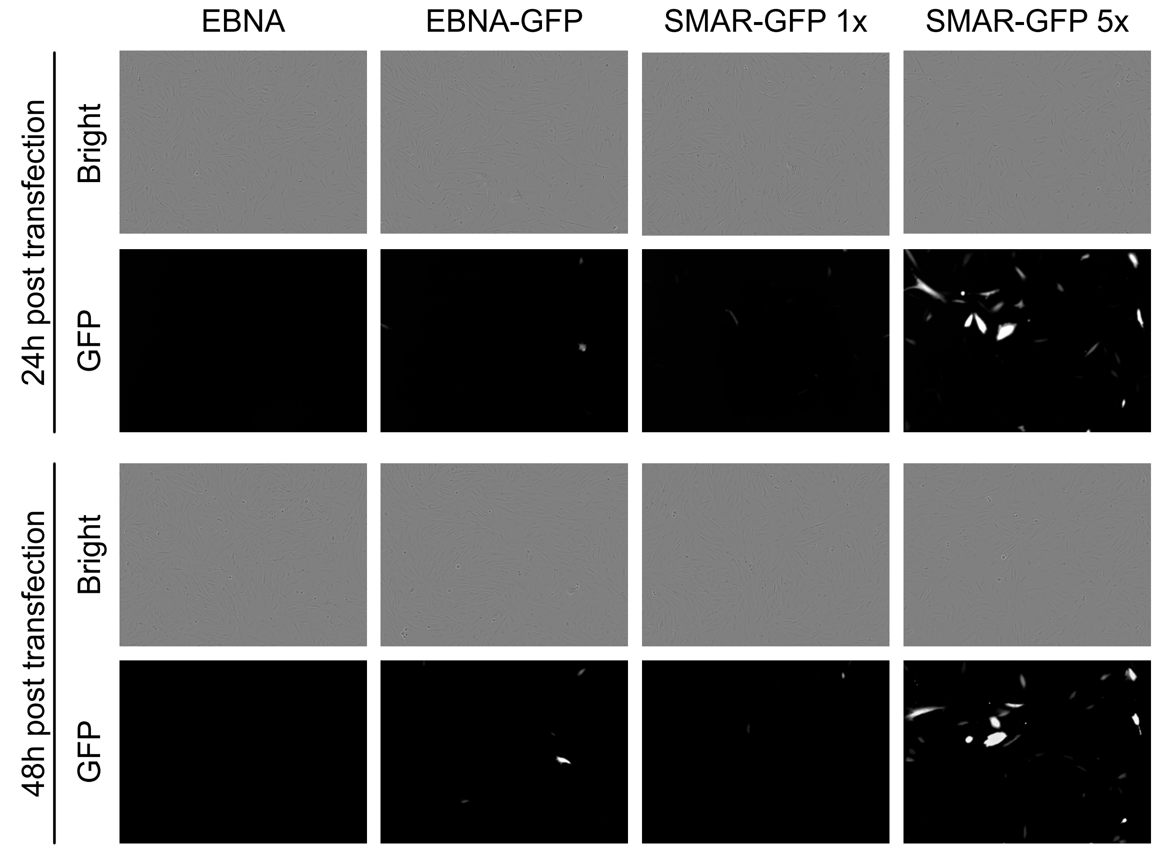

Uncropped, uncorrected brightfield microscopy images used in Figure 2C

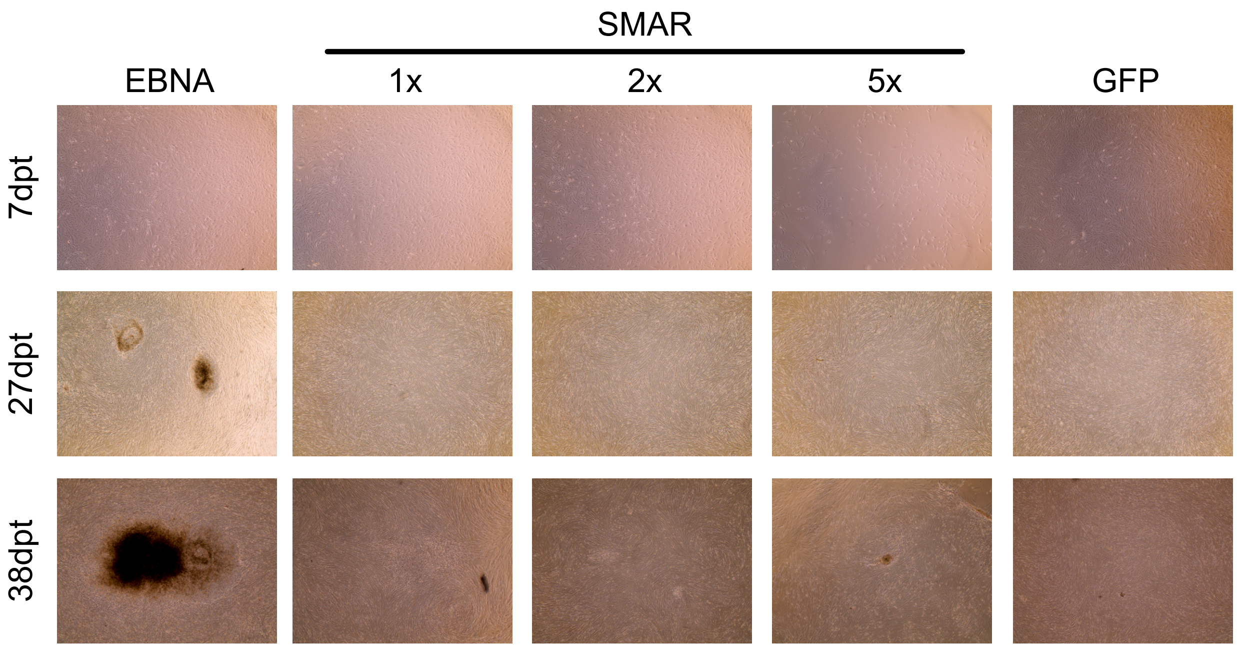

Uncropped, uncorrected fluorescence microscopy images used in Figure 3A

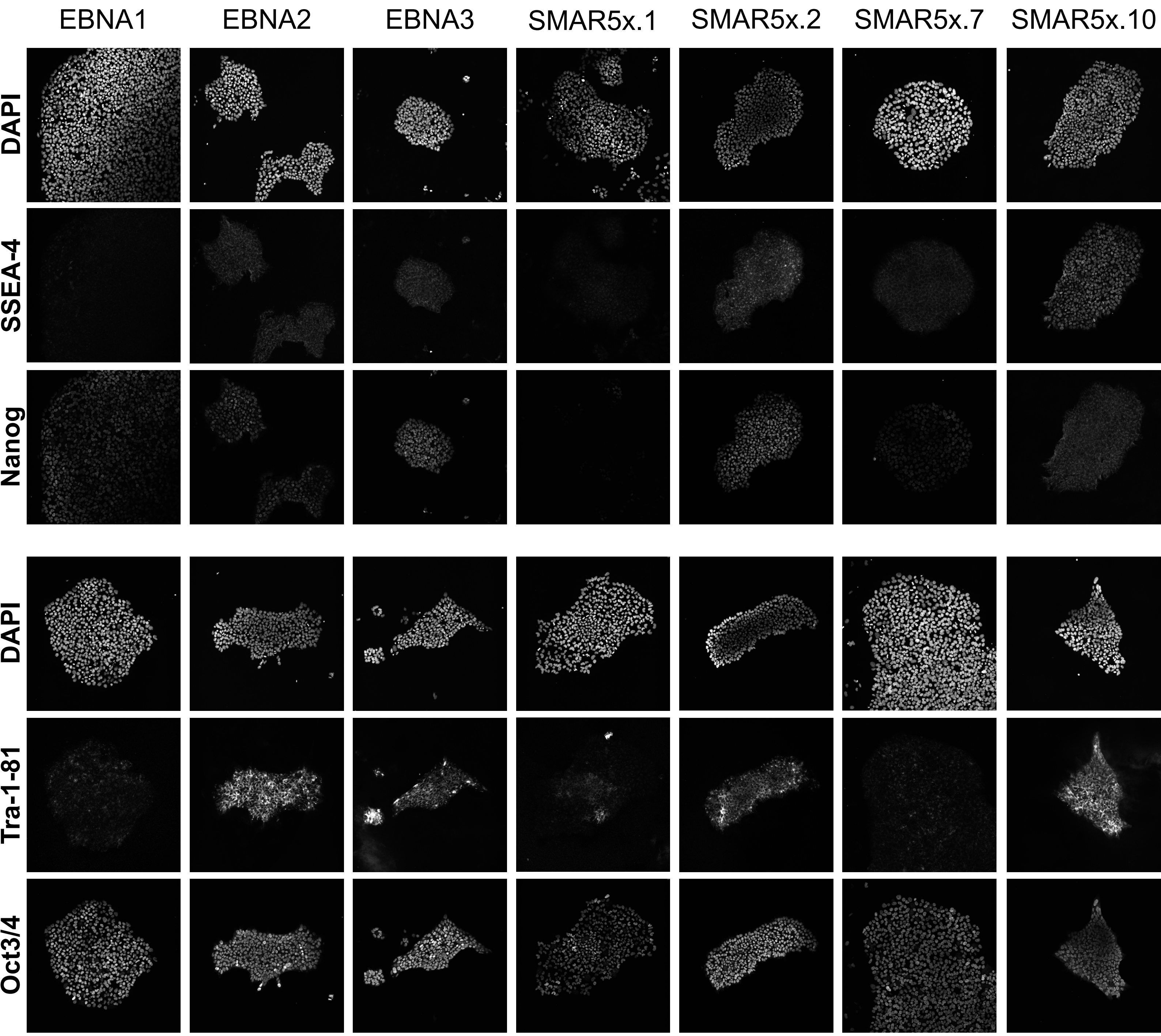

Uncropped, uncorrected fluorescence microscopy images used in Figure 3D

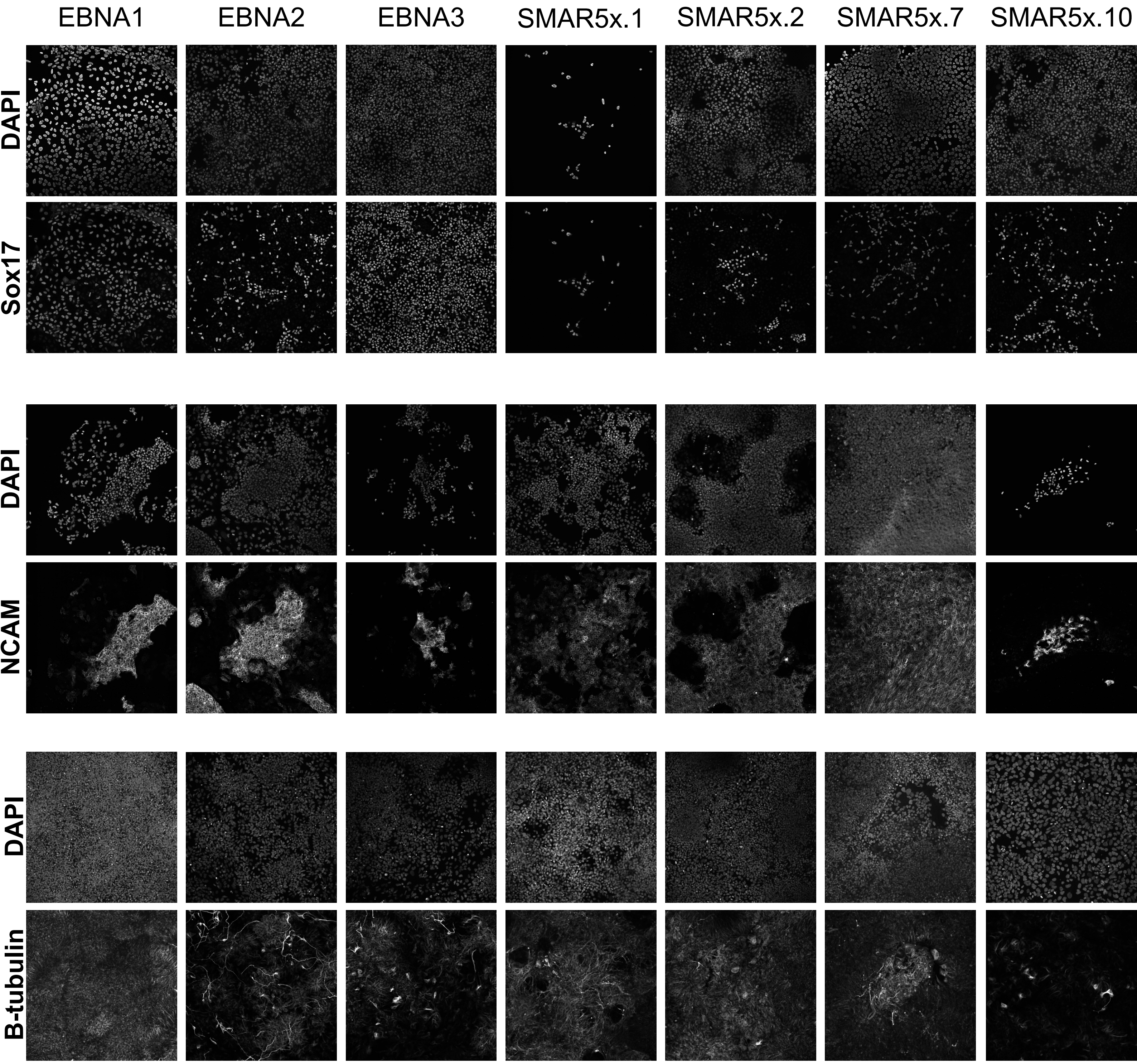

Uncropped, uncorrected brightfield microscopy images used in Figure S1B

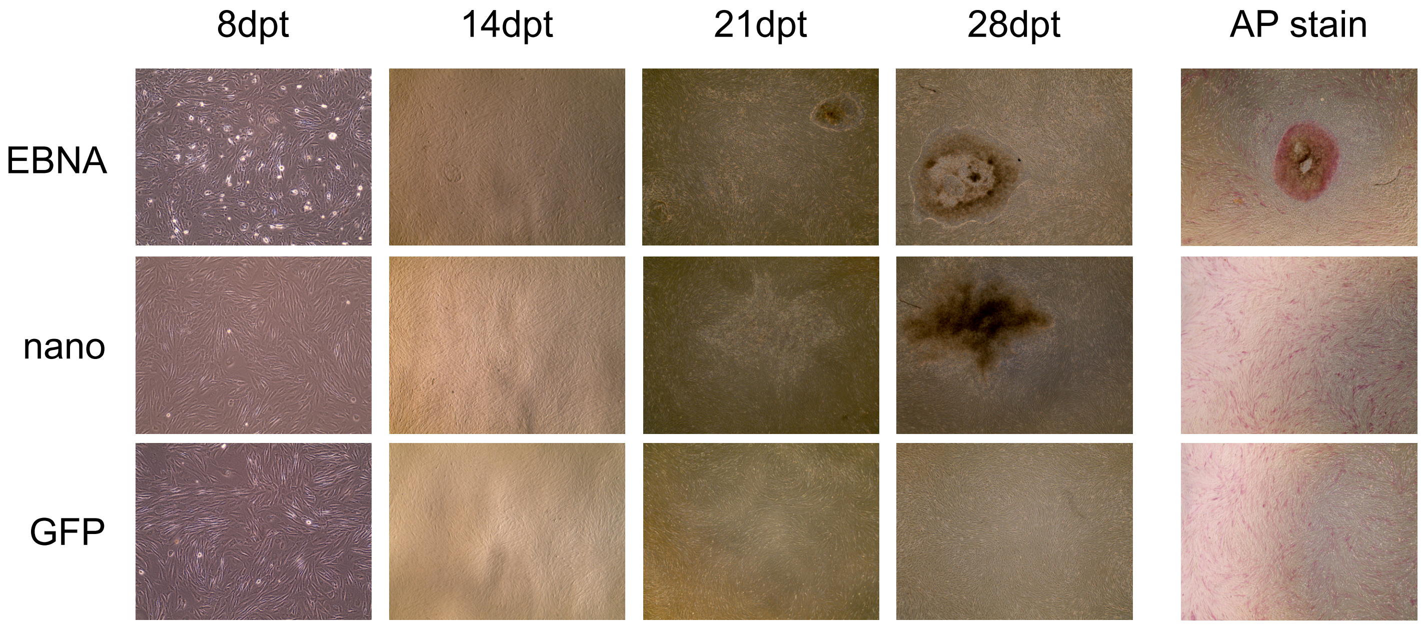

Uncropped, uncorrected brightfield microscopy images used in Figure S2A

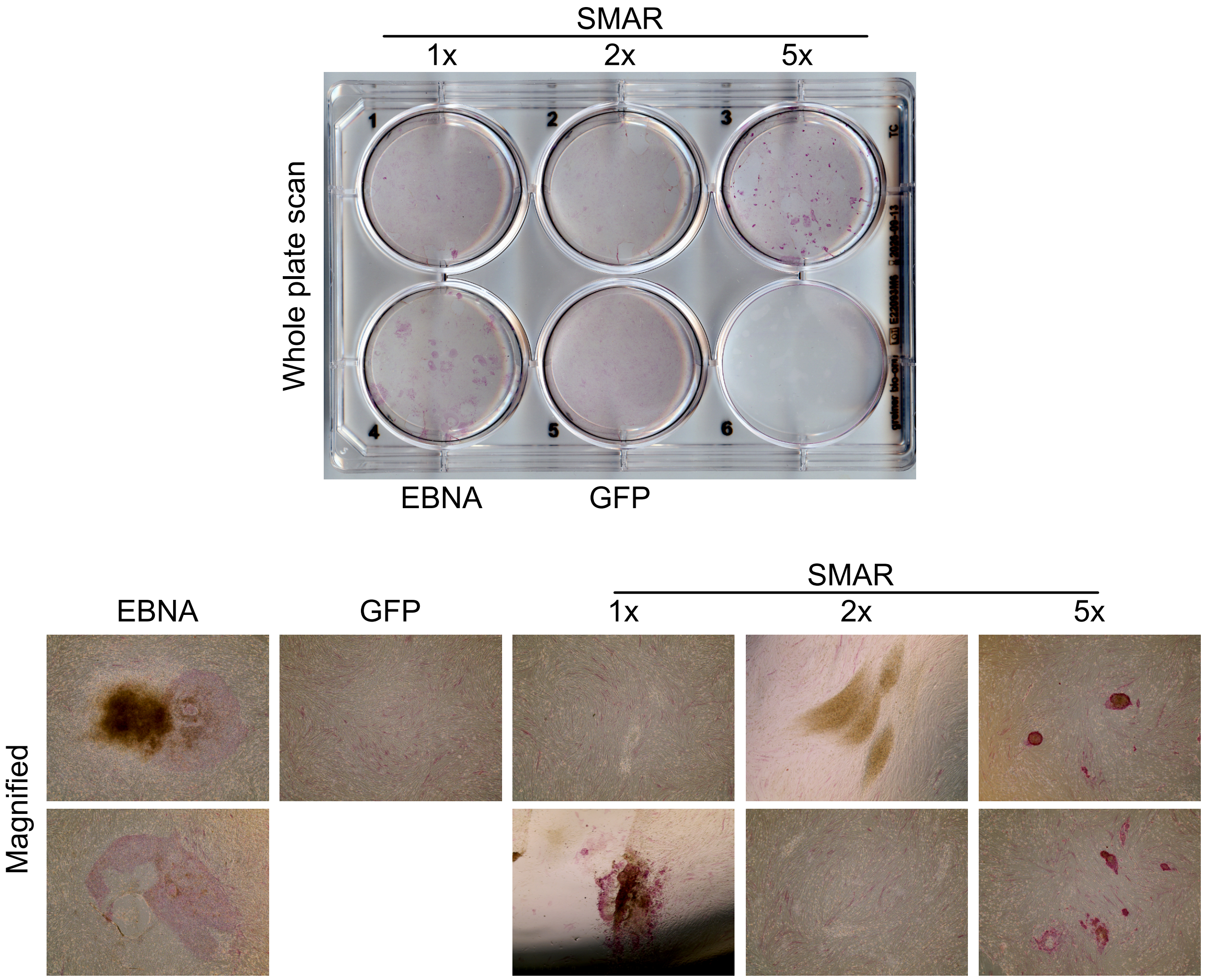

Uncropped, uncorrected brightfield microscopy images used in Figure S2B

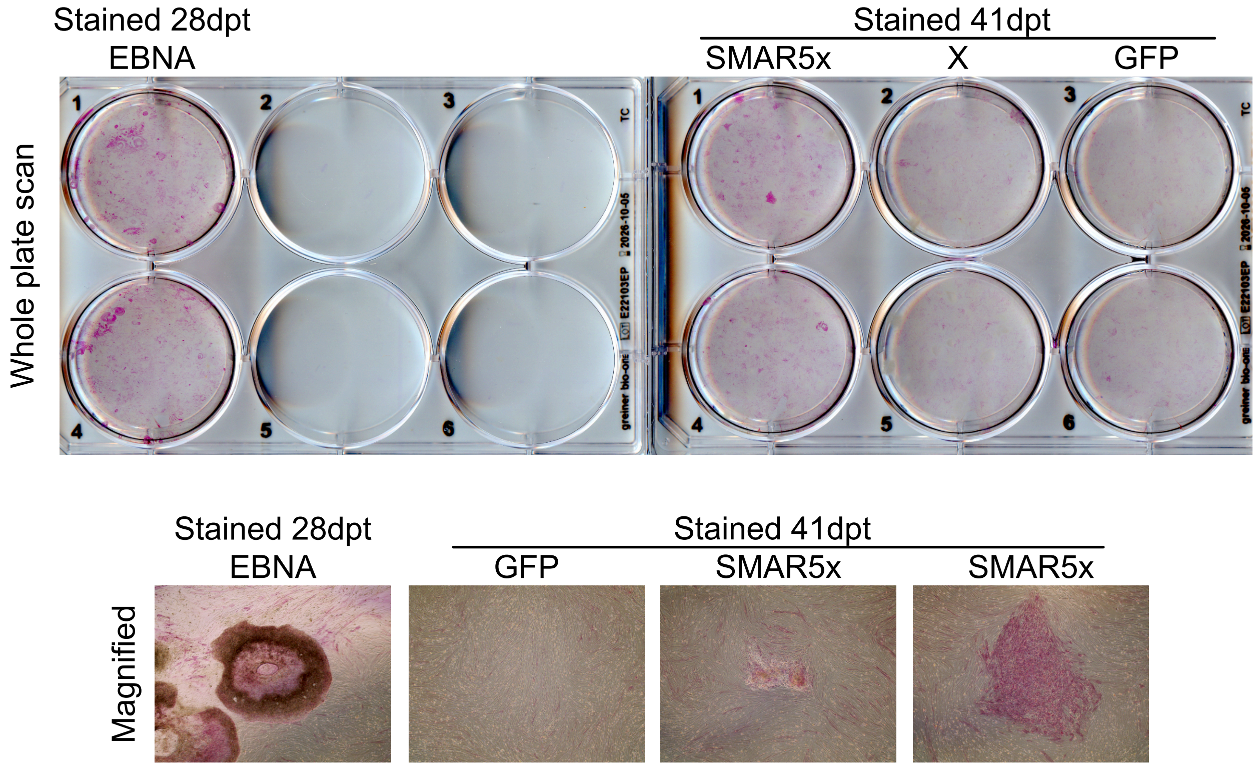

Raw FACS data used in Figure 4

Experiment 1 - iPSC derived NK

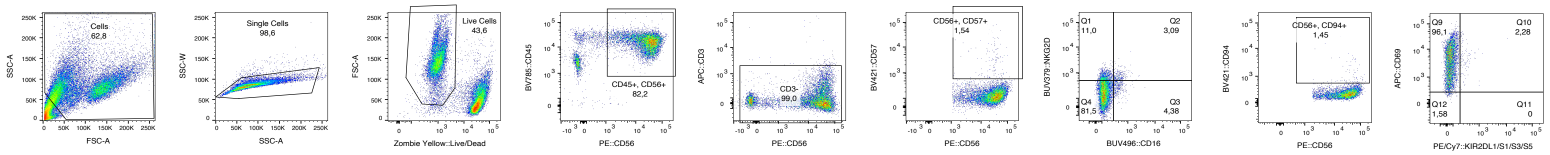

Experiment 1 - blood derived NK

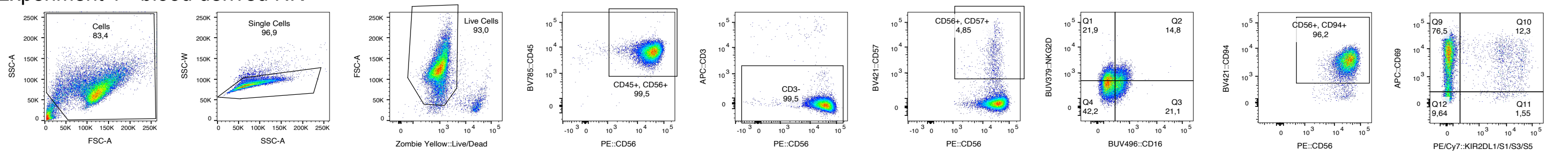

Experiment 1 - iPSC derived NK, unstained

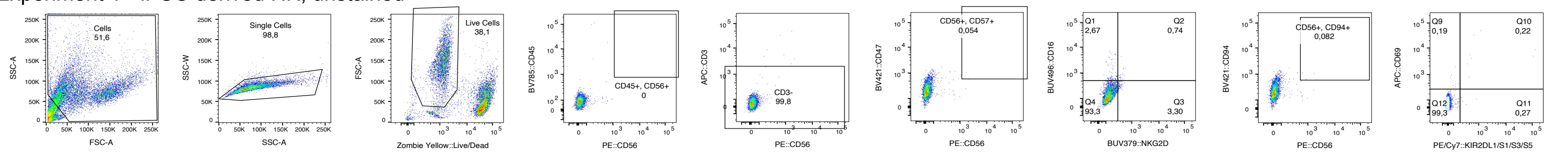

Experiment 1 - blood derived NK, unstained

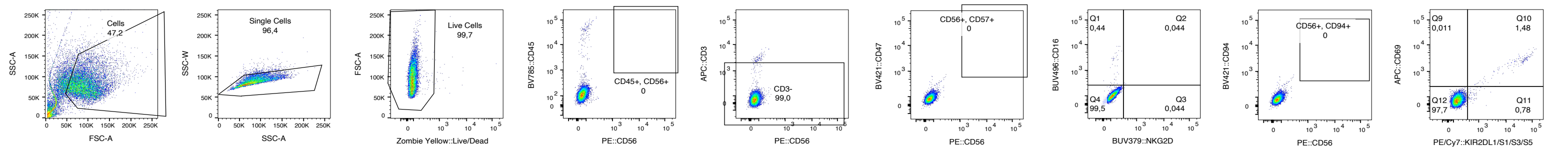

Experiment 2 - iPSC derived NK

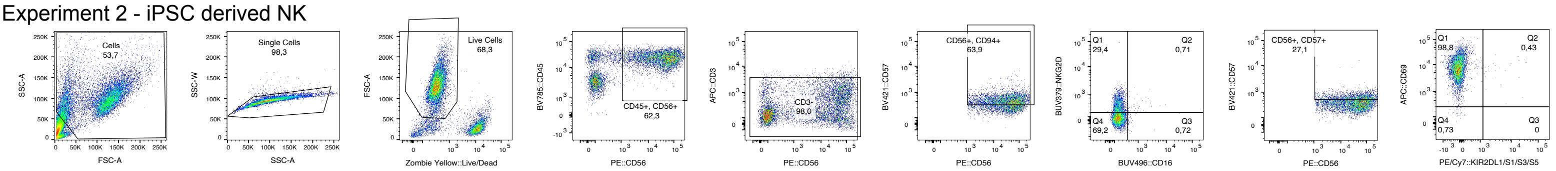

Experiment 2 - blood derived NK

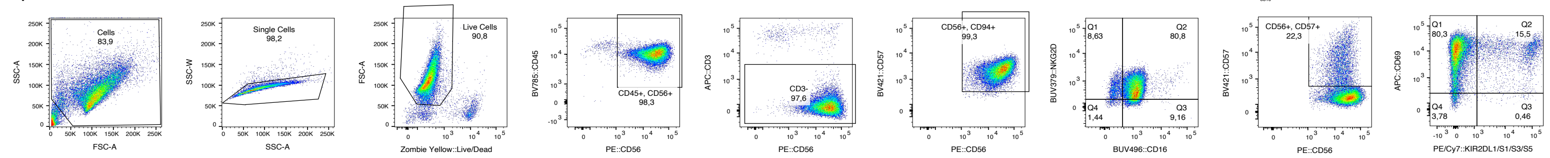

Experiment 2 - iPSC derived NK, unstained

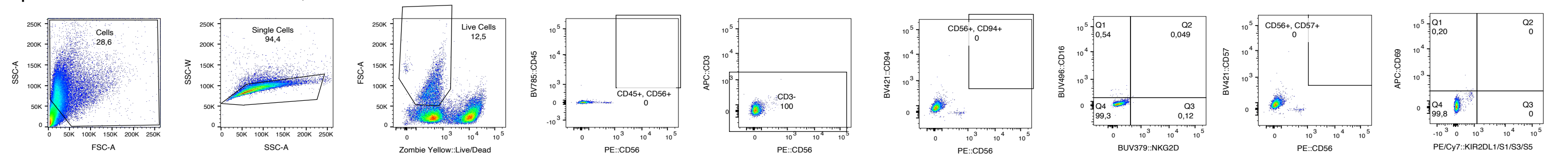

Experiment 2 - blood derived NK, unstained

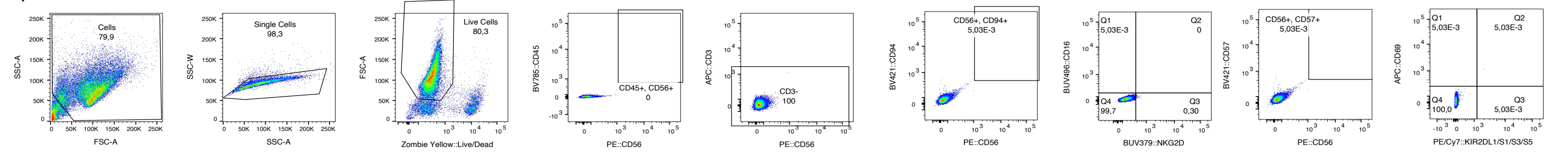

Supplement: Supplementary file 1 [file genes-15-00575-s001.zip › Hartley et al raw data.pdf]
