# Supplementary material for: A Simple Nonviral Method to Generate Human Induced Pluripotent Stem Cells Using SMAR DNA Vectors
Source: Genes (Basel). 2024 Apr 30;15(5):575. doi: 10.3390/genes15050575 (PMC11121542; doi:10.3390/genes15050575)
Supplement: Supplementary file 1 [file genes-15-00575-s001.zip › Supplementary Materials.pdf]

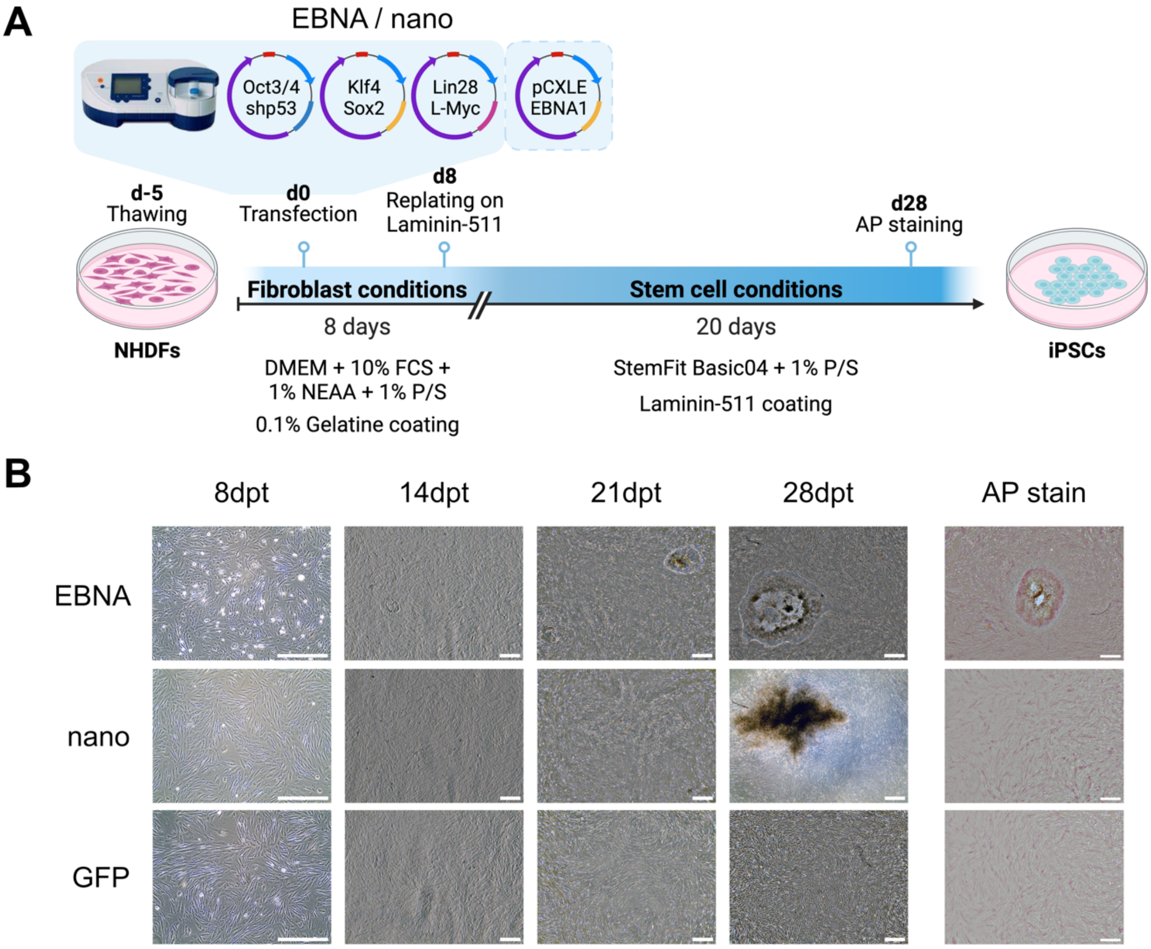

Figure S1 Nanovectors are not capable of fully reprogramming NHDFs without episomal maintenance. **A** NHDFs were transfected with EBNA or nano reprogramming vectors and cultured for 28 days. Cells were then fixed and stained for alkaline phosphatase (AP). **B** Brightfield images of NHDFs after transfection over time. Cells were fixed and stained for alkaline phosphatase (AP) at day 28 (right). Scale = 500µm.

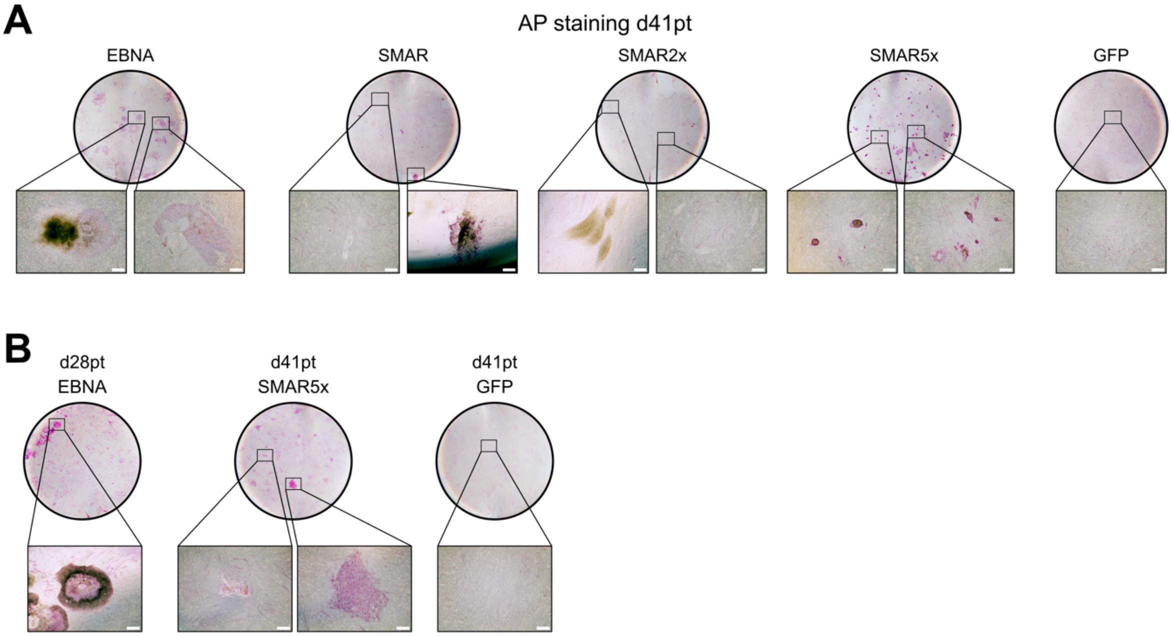

Figure S2 Colonies formed from EBNA and SMAR5x transfection express alkaline phosphatase. NHDFs were transfected with EBNA or SMAR reprogramming vectors and cultured for up to 41 days. Cells were then fixed and stained for alkaline phosphatase (AP). **A** Representative images of AP-stained wells 41 days post transfection, after colony picking, for all transfection conditions. **B** Cells were reprogrammed as above, and AP staining of EBNA-transfected cells at day 28 post transfection was compared with SMAR5x transfection at day 41 post transfection.

Table S 1 List of cloning primers used in this study

#	Name	Sequence (5' -> 3')	Template vector	Final product
1	Oct_fwd	CGCTAGCGCTACCGGTGAATTCGCCCTTCACCATGGC	pCXLE_hO_shP53	SMAR_hO
2	Oct_rev	TATCATCGAGCTCGAGTCATATGACTAGTCCCCGAAGCTTGAATTCG		
3	KS_fwd	CGCTAGCGCTACCGGTGAATTCGCCCTTCACCATGTACAAC	pCXLE_hSK	SMAR_hSK
4	KS_rev	TATCATCGAGCTCGAGTTAAAAATGTCTCTTCATGTGTAAGGCGAGGT		
5	UL_fwd	CGCTAGCGCTACCGGTGAATTCGCCCTTCACCATGGAC	pCXLE_hUL	SMAR_hUL
6	UL_rev	TATCATCGAGCTCGAGTCAATTCTGTGCCTCCGGGAGCA		
7	shP53_fwd	AATGTATCTTACATGGATCCGACGCCGCCA	pCXLE_hO_shP53	SMAR_hO_shP53<br>SMAR_GFP_shP53
8	shP53_rev	CCTTTTGCTCACATGCCCCGGGCTGCAGGA		
9	GFP2AP_fwd	TTTTGGCAAAGAATTCATGCCCGCCATGAAGATCG	pSMARt	EBNA_GFP-p2A-Puro
10	GFP2AP_rev	CCCGAAGCTTGAATTCTCAGGCACCGGGCTTGC		

Table S 2 List of qRT-PCR primers used in this study

Name	Sequence (5' -> 3')	Target gene	Product size (bp)	Purpose
SOX17_F	GCTTTCATGGTGTGGGCTAA	SOX17	105	trilineage differentiation of iPSCs, endoderm
SOX17_ R	CGCCTTCCACGACTTGC			
FOXA2_F	TACAGGCGCAGCTACACGCACGCAAAG	FOXA2	216	trilineage differentiation of iPSCs, endoderm
FOXA2_R	GCGGGGCACCTTCAGGAAACAGTCGT			
KDR_F	CTGGCATGGTCTTCTGTGAAGCA	KDR	790	trilineage differentiation of iPSCs, mesoderm
KDR_R	AATACCAGTGGATGTGATGGCGG			
Brachyury_F	GGATGAAGGCTCCCGTCTC	TBXT	208	trilineage differentiation of iPSCs, mesoderm
Brachyury_R	GCTGTGATCTCCTCGTTCTGATA			
Sox1_F	GGTCAAACGGCCCATGAACGC	SOX1	249	trilineage differentiation of iPSCs, ectoderm
Sox1_R	TCCTTCTTGAGCAGCGTCTTGGTCTT			
hu_PAX6-F	TTTGCCCGAGAAAGACTAGC	PAX6	83	trilineage differentiation of iPSCs, ectoderm
hu_PAX6-R	CATTTGGCCCTTCGATTAGA			
human endo_KLF4-F	TGATTGTAGTGCTTTCTGGCTGGGCTCC	KLF4	397	iPSC phenotyping
human endo_KLF4-R	ACGATCGTGGCCCCGGAAAAGGACC			
human endo_c-MYC-F	GCGTCCTGGGAAGGGAGTTCCGGAGC	MYC	325	iPSC phenotyping
human endo_c-MYC-R	TTGAGGGGCATCGTCGCGGGAGGCTG			
hOCT3/4-S1165	GACAGGGGGAGGGGAGGAGCTAGG	POU5F1	144	iPSC phenotyping
hOCT3/4-AS1283	CTTCCCTCCAACCAGTTGCCCCAAAC			
hSOX2-S1430	GGGAAATGGGAGGGGTGCAAAAGAGG	SOX2	151	iPSC phenotyping
hSOX2-AS1555	TTGCGTGAGTGTGGATGGGATTGGTG			
hNANOG-S	CAGCCCTGATTCTTCCACCAGTCCC	NANOG	309	iPSC phenotyping
hNANOG-AS	CGGAAGATTCCCAGTCGGGTTCACC			
hGDF3-S243	CTTATGCTACGTAAAGGAGCTGGG	GDF3	631	iPSC phenotyping
hGDF3-AS850	GTGCCAACCCAGGTCCCGGAAGTT			
hREX1-RT-S	CAGATCCTAAACAGCTCGCAGAAT	ZFP42	305	iPSC phenotyping
hREX1-RT-AS	GCGTACGCAAATTAAAGTCCAGA			
hFGF4-RT-S	CTACAACGCCTACGAGTCCTACA	FGF4	371	iPSC phenotyping
hFGF4-RT-AS	GTTGCACCAGAAAAGTCAGAGTTG			
hESG1-S40	ATATCCCGCCGTGGGTGAAAGTTC	DPPA5	234	iPSC phenotyping
hESG1-AS259	ACTCAGCCATGGACTGGAGCATCC			
hTERT-S3234	CCTGCTCAAGCTGACTCGACACCGTG	TERT	446	iPSC phenotyping
hTERT-AS3713	GGAAAAGCTGGCCCTGGGGTGGAGC			
GAPDH Forward	GCCAAAAGGGTCATCATCTC	GAPDH	117	iPSC phenotyping
GAPDH Reverse	GGTGGTGCAGGAGGCATT			

Table S 3 Cycling conditions for qRT-PCRs to assay iPSC pluripotency and differentiation

**Cycle step**	**Temperature**	**Time**	**Cycles**
Initial denaturation	95°C	3 min	1
Denaturation	95°C	30 sec	
Annealing	58°C (stem) or 58.3°C (differentiated)	30 sec	40
Extension	72°C	30 sec	
Final denaturation	95°C	1 min	1
Annealing	65°C	1 min	1
Melting curve	65°C – 95°C	0.5°C/sec	1

Table S 4 List of primary antibodies and their dilutions used in this study. All antibodies used target human proteins.

Application	Antibody	Clone	Dilution	Company	Catalogue No.
WB	Goat anti-Oct3/4	n-19	1:100	Santa Cruz Biotech (Texas, USA)	sc-8628
WB	Rabbit anti-Klf4	H-180	1:500	Santa Cruz Biotech	sc-20691
WB	Rabbit anti-Sox2		1:1000	Merck Millipore (Darmstadt, DE)	AB5603
WB	Mouse anti-Lin28	C-9	1:200	Santa Cruz Biotech	sc-374460
WB	Mouse anti-L-Myc		1:1000	Abcam (Amsterdam, NL)	ab167315
WB	Mouse anti-α-tubulin	DM1A	1:5000	Thermo Fisher Scientific (Rockford, USA)	62204
IF, mesoderm	Goat anti-NCAM(CD56) IgG		1:100	R&D Systems (Minneapolis, USA)	AF2408
IF, endoderm	Goat anti-SOX17 IgG		1:100	R&D Systems	AF1924
IF, ectoderm	Mouse anti-β-Tubulin-III IgG	SDL.3D10	1:1000	Sigma-Aldrich (St Louis, USA)	T8660
IF, Stem	Mouse anti- SSEA4	MC813-70	1:75	Abcam	AB16287
IF, Stem	Rabbit anti- NANOG		1:100	Abcam	AB21624
IF, Stem	Mouse anti- TRA1-81 IgM	TRA-1-81	1:150	Abcam	AB16289
IF, Stem	Rabbit anti- OCT4		1:300	Abcam	AB1985
Flow cytometry	Mouse BV785 anti-CD45	HI30	1:50	BioLegend (San Diego, USA)	304047
Flow cytometry	Mouse PE anti- CD56	5.1H11	1:50	BioLegend	362508
Flow cytometry	Mouse APC anti- CD3	SK7	1:50	BioLegend	344812
Flow cytometry	Mouse BV421 anti-CD57	QA17A04	1:100	BioLegend	393325
Flow cytometry	Mouse BUV395 anti-CD314 (NKG2D)	1D11	1:50	BD Biosciences (Erembodegem, BE)	743561
Flow cytometry	Mouse BUV496 anti-CD16	3G8	1:40	BD Biosciences	612944
Flow cytometry	Mouse BV421 anti-CD94	HP-3D9	1:100	BD Biosciences	743948
Flow cytometry	Mouse APC anti- CD69	FN50	1:50	BioLegend	310910
Flow cytometry	Mouse PE/Cy7 anti-CD158 (KIR2DL1/S1/S3/S5)	HP-MA4	1:50	BioLegend	339511

Table S 5 List of secondary antibodies and their dilutions used in this study

Application	Antibody	Conjugation	Dilution	Company	Catalogue No.
WB	Goat anti-Mouse	HRP	1:5000	Jackson Immuno-Research (West Grove, USA)	115-035-044
WB	Donkey anti-Rabbit	HRP	1:10,000	Life Technologies (Bleiswijk, NL)	A16023
WB	Donkey anti-Goat	HRP	1:10,000	Life Technologies	A15999
IF	Donkey anti-Goat IgG (H+L)	Alexa Fluor 488	1:500	Invitrogen (Bleiswijk, NL)	A11055
IF	Goat anti-Mouse IgG (H+L)	Alexa Fluor 546	1:500	Invitrogen	A11003
IF	Goat anti-Rabbit IgG (H+L)	Alexa Fluor 488	1:500	Invitrogen	A11008

Table S 6 List of reprogramming vector series used in this study and their properties.

	**EBNA**	**SMAR**	**nano**
Bacterial sequences and antibiotic selection	Yes	Yes	No
Viral Components	Yes	No	No
Oncogenic	Potentially	No	No
Episomal maintenance	Yes	Yes	No
Vector size	Large	Medium	Small
Safety profile	Low	Medium	Very high
Reprogramming capacity	Yes	Untested	Untested
